# Proton Selective Nanoporous Atomically Thin Graphene Membranes for Vanadium Redox Flow Batteries

**DOI:** 10.1002/adma.202510609

**Published:** 2025-11-12

**Authors:** Pavan Chaturvedi, Peifu Cheng, Saban M. Hus, Matthew Coupin, An‐Ping Li, Jamie Warner, Michael S.H. Boutilier, Piran R. Kidambi

**Affiliations:** ^1^ Department of Chemical and Biomolecular Engineering Vanderbilt University Nashville TN 37212 USA; ^2^ Center for Nanophase Materials Sciences Oak Ridge National Laboratory Oak Ridge TN 37831 USA; ^3^ Walker Department of Mechanical Engineering University of Texas at Austin Austin TX 78712‐1591 USA; ^4^ Department of Chemical and Biochemical Engineering Western University London ON N6A 5B9 Canada; ^5^ Department of Mechanical and Aerospace Engineering University of Florida Gainesville FL 32611 USA

**Keywords:** atomically thin membranes, angstrom‐scale pores, CVD graphene, proton exchange membranes (PEMs), poly‐benzimidazole (PBI), vanadium redox flow batteries (VRFBs)

## Abstract

Angstrom‐scale proton‐selective pores in atomically thin 2D materials present fundamentally new opportunities for advancing proton exchange membranes (PEMs). Vanadium Redox Flow Batteries (VRFBs) for grid‐scale energy storage require PEMs with high areal proton conductance (>1 S cm^−2^) and minimal vanadium ion (VO^2+^) crossover. However, state‐of‐the‐art Nafion 212 membranes (N212 ≈50 µm thick), suffer from persistent VO^2+^ crossover reducing performance and efficiency. Here, a layered PEM is demonstrated, comprising monolayer CVD graphene with Angstrom‐scale proton‐selective pores introduced via Ar plasma, integrated with an ultra‐thin ≈300 nm polybenzimidazole (PBI) layer and sandwiched between two Nafion 211 (25 µm thick) layers. The layered architecture facilitates scalable membrane fabrication by mitigating defects while processing and facile stacking of graphene layers allows stochastic non‐selective defect isolation enabling exceptionally low VO^2+^ crossover (selectivity (H^+^ areal conductance / VO^2+^ permeability) ≈6709 × 10^6^ S min cm^−4^), with proton conductance >8 S cm^−2^. Systematic transport experiments supported by resistance‐based transport modelling elucidate the role of defect size, defect isolation, and sealing, as well as layering/stacking, to enable orders of magnitude (>671× over N212) improvements in selectivity, along with areal proton conductance >8 S cm^−2^. This work highlights the potential of atomic‐scale proton‐selective defect engineering in 2D materials, in conjunction with facile stacking and layering of materials as strategies for scalable, high‐performance advances in PEMs for energy, electrochemical, and separation applications beyond VRFBs.

## Graphical abstract

A scalable high‐performance proton‐exchange‐membrane architecture is demonstrated that integrates monolayer CVD graphene with Ar plasma‐engineered Angstrom‐scale proton‐selective pores, stacked to mitigate non‐selective defects, and interfaced with an ultrathin (≈300 nm) polybenzimidazole layer sandwiched between two Nafion 211 sheets. The layered architecture facilitates facile fabrication and stochastically isolates macroscopic defects through the stacking of two individual graphene layers—yielding exceptionally low VO^2+^; permeability and record‐high membrane selectivity.

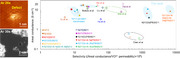

## 1 Introduction

The global transition toward a decarbonized energy infrastructure necessitates scalable, long‐duration energy storage systems capable of buffering the intermittency inherent to renewable sources such as solar and wind. Among the various technologies, vanadium redox flow batteries (VRFBs) have gained traction as a scalable and robust solution for long‐duration stationary grid‐scale energy storage, owing to their non‐flammability, long cycle life, flexible power‐energy scaling, and high operational efficiency.^[^ ^1^, ^2^, ^3^, ^4^ ^]^ Central to VRFB performance is the proton exchange membrane (PEM), which must simultaneously exhibit high proton (H⁺) conductivity and chemical stability while minimizing vanadium ion (VO^2+^, hydrated diameter ≈0.8 nm)^[^ ^5^ ^]^ crossover to mitigate capacity fading and efficiency loss over extended charge‐discharge cycling.^[^ ^6^, ^7^, ^8^, ^9^, ^10^, ^11^, ^12^ ^]^

State‐of‐the‐art PEMs such as Nafion are widely used in VRFBs due to their high proton conductance (≈1–20 S cm^−^^2^)^[^ ^13^ ^]^ and excellent chemical and mechanical stability, but suffer from VO^2+^ crossover.^[^ ^14^, ^15^, ^16^, ^17^ ^]^ This crossover disrupts the charge balance and water distribution between electrolyte tanks—posing a fundamental challenge to the long‐term reliability and economic viability of VRFBs. Various strategies have been pursued to mitigate these losses, including the development of alternative polymer membranes and membrane materials e.g., polybenzimidazole (PBI),^[^ ^18^, ^19^, ^20^, ^21^, ^22^, ^23^ ^]^ sulfonated polyetheretherketone (SPEEK),^[^ ^24^, ^25^, ^26^ ^]^ modifying Nafion membranes^[^ ^9^, ^10^, ^27^, ^28^, ^29^, ^30^ ^]^ via interfacial coatings, and hybrid composites incorporating materials including flakes of graphene oxide (GO),^[^ ^26^, ^28^, ^29^, ^30^, ^31^ ^]^ 2D materials,^[^ ^32^, ^33^, ^34^ ^]^ zeolites,^[^ ^35^ ^]^ among others.^[^ ^14^, ^36^, ^37^, ^38^ ^]^ While these approaches suppress crossover, they frequently compromise H⁺ conductivity due to narrowing of channels, which increases the mass transport resistance in addition to swelling of the polymers that may further alter the polymer and fillers interface i.e., resulting in poor nano‐structural control, and interfacial defects—reinforcing the long‐standing trade‐off between selectivity and conductivity.^[^ ^14^, ^38^ ^]^ This decrease in proton conductivity is equivalent to increasing the thickness of Nafion membranes, which results in increased voltage losses due to ohmic drop. Hence, the development of a thinner membrane with high proton conductivity and exhibiting very low VO^2+^ ion crossover is desirable.

Atomically thin 2D materials offer a fundamentally different route to circumvent this trade‐off.^[^ ^39^, ^40^, ^41^, ^42^ ^]^ The pristine 2D lattice of monolayer graphene is impermeable to He atoms as well as hydrated ions (here, VO^2+^ ions) but allows electric‐field‐driven permeation of protons, albeit with low conductance of ≈3 mS cm^−2^ (in 0.1 M HCl), which is significantly lower than Nafion ≈1–20 S cm^−2^.^[^ ^39^, ^43^ ^]^ Angstrom‐scale defects introduced into the 2D lattice can enhance proton transport through atomically thin 2D materials such as graphene while excluding ions like VO^2+^ ≈0.8 nm. Monolayer graphene synthesized via chemical vapor deposition (CVD) inherently possesses such sub‐nm defects from bottom‐up synthesis,^[^ ^44^, ^45^, ^46^ ^]^ and additional proton‐selective pores can be introduced via ex situ or in situ methods such as energetic ion bombardment, plasma treatment, as well as kinetic or temperature control of CVD processes, respectively.^[^ ^47^, ^48^, ^49^, ^50^, ^51^, ^52^, ^53^ ^]^ However, realizing scalable PEMs based on atomically thin graphene remains challenging, as fabrication‐induced defects such as large‐cracks/tears can severely degrade membrane selectivity via non‐selective leakage of non‐specific ions (*e.g*. VO^2+^ ions).^[^ ^54^, ^55^, ^56^, ^57^ ^]^

Here, we demonstrate a layered PEM architecture for VRFBs that leverages unique transport properties of atomically thin CVD graphene while systematically eliminating leakage pathways. Our optimized membrane comprises two stacked monolayers of Ar plasma‐treated CVD graphene each featuring Angstrom‐scale pores, interfaced with a thin (≈300 nm) PBI layer, and sandwiched between two sheets of Nafion 211. This design facilitates scalable fabrication by stochastic isolation of non‐selective defects and tears via stacking two monolayers of CVD graphene and reduces macroscopic leakage pathways by interfacing with PBI. The resulting N211|Gr‐Ar 30 s|Gr‐Ar 30 s|PBI|N211 membranes achieve negligibly low VO^2+^ permeability, with a selectivity (H^+^ areal conductance/VO^2+^ permeability) of ≈6709 × 10⁶ S min cm^−^⁴ and H⁺ conductance >8 S cm^−^^2^, representing over 671× improvement in selectivity relative to commercial state‐of‐the‐art Nafion 212 membranes with similar overall thickness. Our findings present a new design paradigm for high‐performance PEMs by combining atomic‐scale defect engineering with strategic material layering and stacking, to simultaneously achieve high selectivity and conductivity, thus overcoming the conventional trade‐off that has constrained PEMs for VRFBs.

## 2 Results and Discussion

### 2.1 Fabrication of Centimeter‐Scale Atomically Thin Graphene Membranes on PCTE Support and Angstrom‐Scale Proton Selective Pore Creation via Ar Plasma

**Figure 1:**
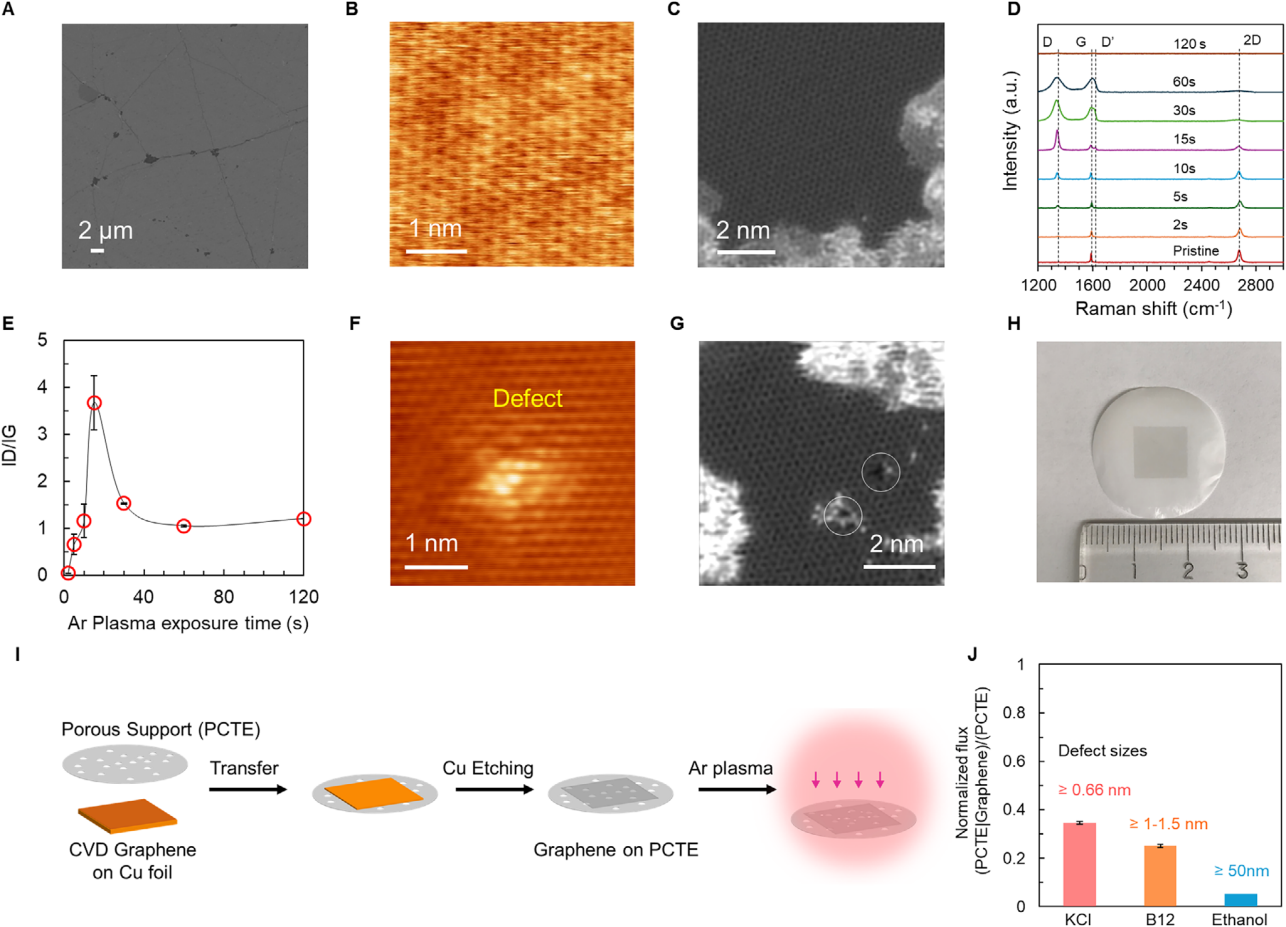
Fabrication of centimeter‐scale nanoporous atomically thin graphene membranes (NATMs). A) SEM and B) atomic resolution scanning tunnelling microscopy (STM) image of CVD graphene on Cu foil. C) Atomic resolution scanning transmission electron microscopy (STEM) image of as synthesized CVD graphene after transfer to TEM grids. D) Raman spectra for CVD graphene transferred on to 300 nm SiO_2_/Si wafer before and after Ar plasma exposure. Ar plasma is performed on graphene after transfer to SiO_2_/Si. Increasing exposure to Ar plasma results in an increase in the D and D’ defect peaks. E) I_D_/I_G_ plot with increasing Ar plasma exposure time computed from Raman spectra in (D). F) STM image of CVD graphene on Cu after 20s of Ar plasma exposure. Also see Figure S2 (Supporting Information) for additional STM images. G) STEM images of proton‐selective sub‐nanometer effects/pores (white circle) introduced into the lattice of CVD graphene via exposure to 30s Ar plasma before transfer to TEM grids for imaging. Also see Figure S1 (Supporting Information). H) Optical image of the centimeter‐scale CVD graphene (region with darker contrast) transferred on PCTE supports (pore diameter ≈200 nm) with transfer process shown schematically in (I). J) Normalized flux ((PCTE | Graphene) / PCTE) for KCl (hydrated diameter of K^+^ and Cl^−^ ≈0.66 nm), vitamin B12 (≈1.0–1.5 nm), and ethanol. Pressure‐driven ethanol transport probes large tears and defects ≥50 nm, while diffusion‐driven transport of KCl and B12 probes defect sizes ≥0.66 nm and ≥1.0–1.5 nm, respectively over centimeter‐scale CVD graphene areas.

**Figure 2:**
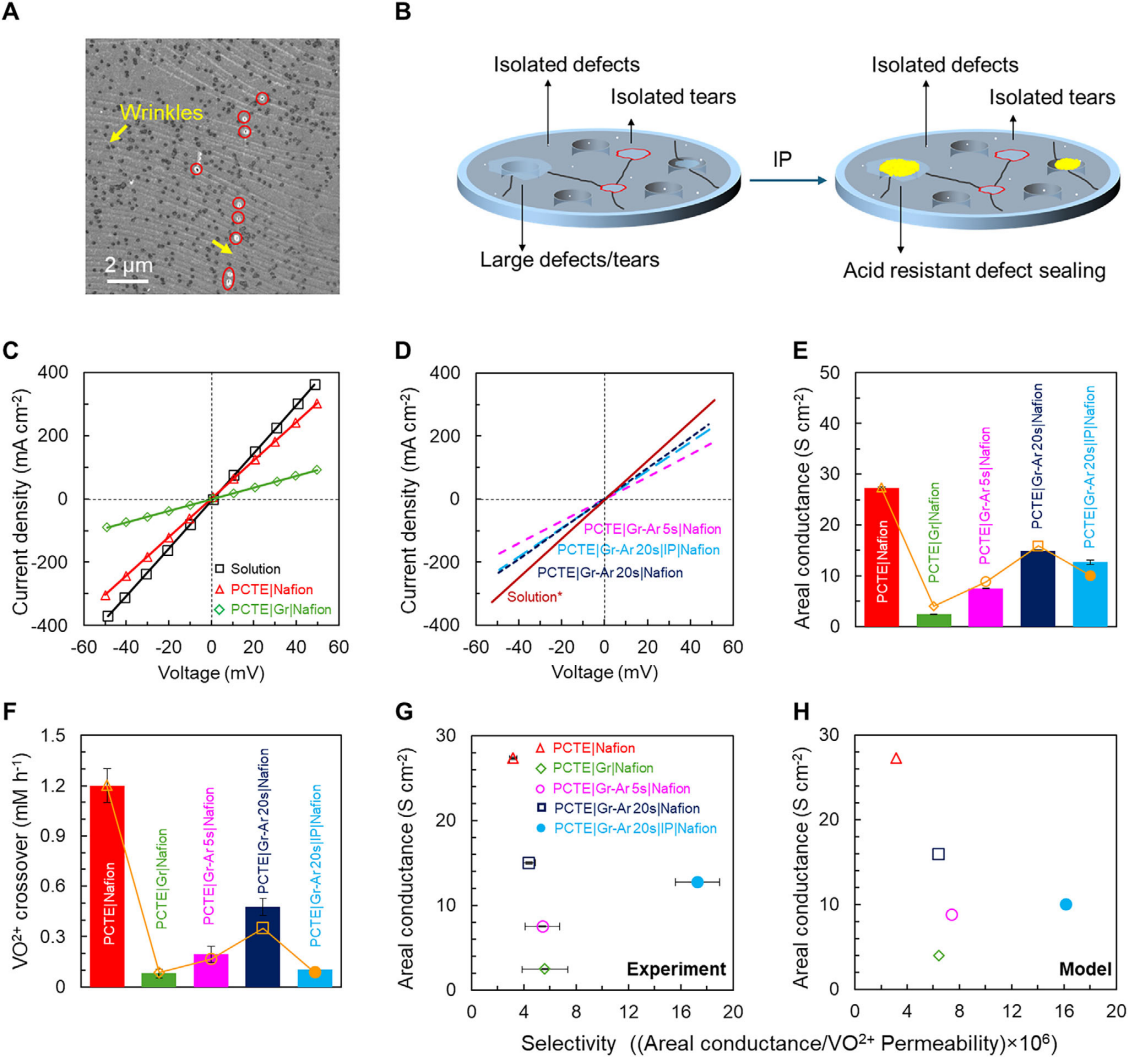
Transport characteristics of nanoporous atomically thin graphene membranes on PCTE supports. A) SEM image of graphene transferred on poly‐carbonate‐track‐etched (PCTE, pore diameter ≈200 nm). Red circles show tears and damage to graphene on PCTE pores that seem to align along wrinkles in graphene (marked with yellow arrows). B) Schematic of sealing tears and damage in graphene via acid‐resistant interfacial polymerization (IP). C,D) *I–V* curves measured in 2 M H_2_SO_4_ for graphene on PCTE with and without Ar plasma treatment followed by defect sealing and in‐flliing with Nafion. E) Areal conductance plots computed from *I–V* curves in (C) and (D) after solution resistance correction (see Table S1, Supporting Information). F) VO^2+^ crossover measured using 1 M VOSO_4_ + 2 M H_2_SO_4_ in the feed side and 1 M MgSO_4_ + 2 M H_2_SO_4_ in the permeate side for the graphene membranes. Yellow symbols on the data bars indicate computed values of crossover using the resistance‐based transport model (see Experimental Section). G) Experimentally measured areal conductance vs selectivity (ratio of areal H^+^ conductance (S cm^−2^) and permeability of VO^2+^ ions (cm^2^ min^−1^), results in units S min cm^−4^) plot for the graphene membranes, and H) corresponding computed values using the resistance‐based transport model show good agreement. The defect‐sealed graphene membranes show higher selectivity while maintaining adequate proton conductance for practical applications.

Atomically thin membrane applications necessitate large‐area 2D material synthesis and catalytic CVD has emerged as the preferred route for scalable cost‐effective bottom‐up synthesis of high‐quality graphene.^[^ ^58^, ^59^, ^60^ ^]^ **Figure** **1** shows the characterization of as‐grown synthesized CVD graphene and after exposure to argon plasma, while **Figure** **2** shows the corresponding ionic transport characteristics. SEM image of CVD graphene on Cu foil with characteristic features such as wrinkles (Figure 1A) indicates the presence of a complete film as well as minor regions of darker contrast consistent with multilayer domains underneath the continuous monolayer CVD graphene film. Scanning tunnelling microscopy (STM, Figure 1B) of CVD graphene on Cu foil and scanning transmission electron microscopy STEM (Figure 1C, also see Figure S1, Supporting Information) after transfer to TEM grids confirm the presence of a hexagonal honeycomb lattice indicating high crystallinity of the CVD graphene films. Raman spectroscopy for CVD graphene transferred to 300 nm SiO_2_/Si wafer (Figure 1D) shows the characteristics G peak ≈1590 cm^−1^ (FWHM ≈10 cm^−1^), 2D peak ≈2680 cm^−1^ (FWHM ≈ 30 cm^−1^), I_2D_/I_G_ >1 and the absence of D‐peak confirms high‐quality monolayer CVD graphene.^[^ ^61^, ^62^ ^]^ To introduce proton‐selective defects in the graphene lattice, CVD graphene was subjected to Ar plasma (mid power ≈11 W, chamber pressure ≈500 mTorr) for varying times (see Experimental Section). Raman spectra (Figure 1D, also see Figure S4, Supporting Information for Ar plasma at high power ≈30 W) evidence the evolution of D and D’ peak, suggesting introduction of lattice defects with increasing plasma time. The corresponding I_D_/I_G_ ratios (Figure 1E) computed from the Raman spectra also show an increase with increasing Ar plasma time with a peak ≈20 s. STM images (Figure 1F; Figure S2, Supporting Information) and STEM (Figure 1G; Figure S1C–G, Supporting Information including size distribution) further confirm the introduction of Angstrom‐scale defects into the CVD graphene lattice via Ar plasma exposure.

To evaluate transport characteristics of the intrinsic defects inherently present in the high‐quality CVD graphene using a combination of diffusion‐driven transport with analytes of different sizes (≈0.66–1.5 nm) as well as pressure‐driven transport with ethanol (defects >50 nm), we transfer CVD graphene on to polycarbonate track etched (PCTE, pore diameter ≈200 nm) supports (see Figure 1H–J).^[^ ^50^, ^52^, ^57^, ^58^, ^59^, ^63^, ^64^, ^65^, ^66^, ^67^, ^68^, ^69^, ^70^ ^]^ Figure 1H shows an optical image with a dark square of uniform contrast in the center of a circular PCTE disc indicating successful centimeter‐scale CVD graphene transfer. The straight and uniform pores of PCTE supports allow for accurate transport measurements with robust statistics and porosity ≈10% ensures minimal crosstalk from pore overlap.

Based on the geometry and scaling laws of CVD graphene on PCTE supports, a single ≈4 nm pore in CVD graphene would offer similar resistance to that of a PCTE pore (≈200 nm diameter and ≈10 µm length) in diffusion‐driven transport, while for pressure‐driven flow a ≈50 nm pore in CVD graphene would have the same resistance as the PCTE support pore.^[^ ^50^, ^58^, ^64^, ^66^, ^67^, ^68^ ^]^ Figure 1J shows normalized flux (flux of PCTE+Graphene/flux of PCTE) for pressure‐driven transport of ethanol ≈5%, indicating the presence of large tears or damages (≥50 nm) from transfer along wrinkles (see SEM image of large defects in Figure 2A) over ≈5% area of CVD graphene and ≈95% CVD graphene coverage on PCTE supports.^[^ ^64^ ^]^ Normalized diffusive flux of KCl (hydrated diameter ≈0.66 nm) is ≈35% and vitamin B12 (diameter ≈ 1–1.5 nm) is ≈25%. The difference of ≈10% between B12 and KCl normalized flux suggests ≈10% defects have sizes between 0.66 and 1 nm (Figure 1J). These diffusive transport measurements are further corroborated via facile acid etch test (see Figure S3, Supporting Information) of the Cu underneath intrinsic defects in CVD graphene and are fully consistent with prior reports.^[^ ^57^, ^69^ ^]^

Next, the PCTE|Gr membranes were coated with Nafion (see methods) to measure transport of protons (H^+^) via *I–V* measurements (Figure 2C) using a custom‐built H‐cell (see Figure S5, Supporting Information). The areal resistance of the membranes is calculated from the slope of the *I–V* curves and a correction for the solution resistance (see Table S1, Supporting Information) provides the membrane resistance. The inverse of the membrane resistance normalized to area provides the areal conductance for PCTE+Gr+Nafion membranes ≈2.4 S cm^−2^ (Figure 2E). The corresponding diffusive VO^2+^ crossover ≈0.08 mm h^−1^ (measured by analyzing the UV–vis spectroscopy of diffused VO^2+^ ions, see methods and Figure S7, Supporting Information) is shown in Figure 2F. The control membrane (PCTE|Nafion) exhibits a proton conductance of ≈27.3 S cm^−2^ with a VO^2+^ crossover of ≈ 1.2 mm h^−1^. The PCTE+Gr+Nafion membranes hence show a noteworthy reduction of ≈91% in areal conductance with ≈93% reduction in VO^2+^ ion crossover for pristine compared to controls PCTE+Nafion, respectively.

To selectively increase the proton transport without increasing the crossover, CVD graphene (on PCTE) was subjected to Ar plasma (see Experimental Section) with varying time to introduce defects as shown in Figure 1D–G. However, attempts to selectively increase areal conductance by introducing Angstrom‐scale defects via exposure to Ar plasma, also resulted in increased VO^2+^ crossover (Figure 2E,F) e.g., 5 s Ar plasma exposure (PCTE|Gr‐Ar 5 s |Nafion) increases areal conductance to ≈7.5 S cm^−2^ (≈300% increase from PCTE|Gr|Nafion), while VO^2+^ crossover also increases to ≈0.194 mm h^−1^ (≈250% increase from PCTE|Gr|Nafion). Similarly, 20 s Ar plasma exposure (PCTE|Gr‐Ar 20 s|Nafion) increased areal conductance to ≈15 S cm^−2^ (≈520% increase from PCTE|Gr|Nafion) and VO^2+^ crossover to ≈0.48 mm h^−1^ (≈600% increase from PCTE+Gr+Nafion). A similar percentage increase in areal conductance and crossover despite the formation of Angstrom‐scale pores in the 2D lattice indicates the introduced defects in combination with pre‐existing intrinsic defects as well as large defects/tears from fabrication (Figure 2A) are providing VO^2+^ leakage pathways.

Sealing the fabrication induced large defects/tears (Figure 2B) via acid resistant interfacial polymerization (IP) using branched‐poly ethyleneimine and cyanuric acid (see Figure S6, Supporting Information and Experimental Section) for the 20 s Ar plasma exposed membrane, followed by infilling with Nafion (PCTE|Gr‐Ar 20 s|IP|Nafion) indeed allows for a notable decrease in VO^2+^ crossover to ≈0.10 mm h^−1^ (≈480% reduction from PCTE|Gr‐Ar 20 s|Nafion ≈0.48 mm h^−1^) with areal conductance ≈13 S cm^−2^ (≈13% lower than PCTE|Gr‐Ar 20 s|Nafion ≈15 S cm^−2^). Figure 2G shows an increase in selectivity (H^+^ areal conductance/VO^2+^ permeability) ≈17.3 × 10^6^ S min cm^−4^ for the PCTE|Gr‐Ar 20 s|IP|Nafion membrane after sealing large defects via IP process while retaining areal conductance ≈13 S cm^−2^. Resistance‐based transport modelling (see Figure S8 and Table S2, Supporting Information) further indicates that sealing of non‐selective large tears/defects increases membrane selectivity (Figure 2I) in agreement with the experimental observations (Figure 2G).

### 2.2 Scalable Integration of Atomically Thin CVD Graphene with Nafion 212 for PEMs and Facile Stacking of Graphene Layers for Stochastic Defect Isolation

**Figure 3:**
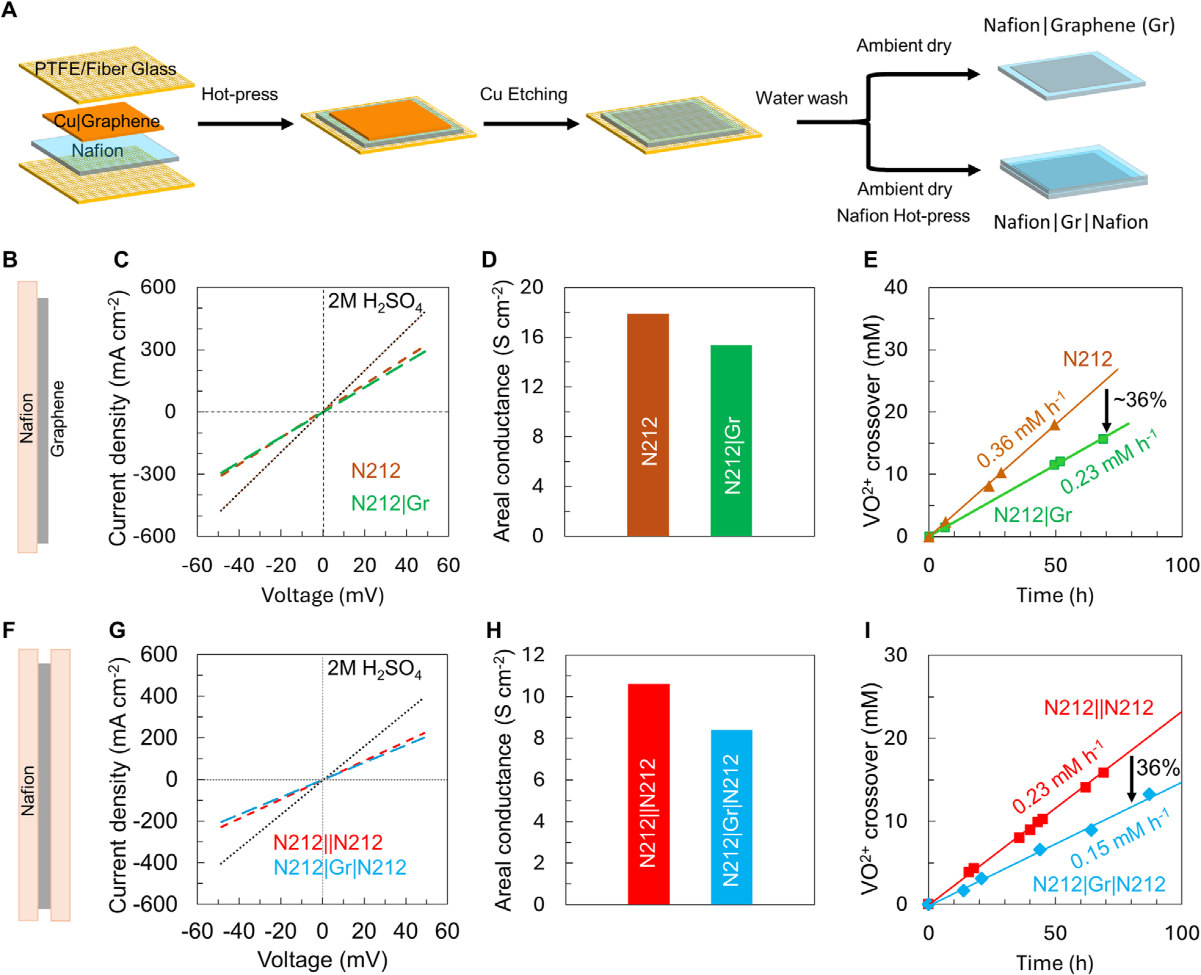
Integrating monolayer CVD graphene with state‐of‐the‐art PEM – Nafion 212. A) Schematic of Nafion ‐ graphene composite PEM fabrication. Two different membrane geometries were fabricated with Nafion 212 (N212, ≈50 µm thick): B) N212|Gr and F) N212|Gr|N212 sandwich membranes. *I–V* curves for C) N212|Gr, and G) N212|Gr|N212, measured in 2 M H_2_SO_4_ and corresponding areal conductance in (D) and (H), respectively, computed from the *I–V* curves after solution resistance correction (see Table S3, Supporting Information). The dotted black line in C and G is *I–V* curve for 2 M H_2_SO_4_ solution. VO^2+^ crossover as a function of time for E) N212|Gr, and I) N212|Gr|N212 sandwich membranes measured using 1 M VOSO_4_ + 2 M H_2_SO_4_ in the feed side and 1 M MgSO_4_ + 2 M H_2_SO_4_ in the permeate side.

Having characterized transport characteristics through intrinsic defects as well as defects formed via Ar plasma in graphene using PCTE supports, we proceed to explore scalable integration with state‐of‐the‐art Nafion 212 membranes. We fabricate two distinct geometries ‐ N212|Gr and N212|Gr|N212 sandwich membranes (**Figure** **3**A, also see methods) and measure areal proton conductance (Figure 3D,H) and VO^2+^ crossover (Figure 3E,I), respectively.

*I–V* curves measured in 2 M H_2_SO_4_ electrolyte for only solution (no membrane), controls (N212 and N212||N212) and graphene membranes (N212|Gr and N212|Gr|N212) are used to compute areal conductance (Figure 3C,G). The integration of CVD graphene results in areal proton conductance of ≈15.3 S cm^−2^ for N212|Gr, in comparison to ≈17.9 S cm^−2^ for N212(Figure 3D). The corresponding conductivity values for N212 and N212|Gr are ≈90.3 and 75.1 mS cm^−1^ respectively (Table S3, Supporting Information), which are well within the expected range of ≈100 mS cm^−1^ for N212 at 100% RH. Similarly, N212|Gr|N212 sandwich membrane exhibits areal proton conductance ≈8.4 S cm^−2^ in comparison to ≈10.6 S cm^−2^ for N212||N212(Figure 3H). The corresponding conductivity values for N212||N212 and N212|Gr|N212 are ≈105.8 and 84 mS cm^−1^, respectively (Table S3, Supporting Information). Addition of graphene results in ≈14% reduction (for N212) and ≈20% reduction (for sandwich geometry) in proton conductance, reflecting minor hindrance to proton transport with the addition of graphene. However, the significantly higher proton conductance compared to reported values of pristine graphene ≈3 mS cm^−2^ suggest the role of intrinsic and fabrication‐induced defects, consistent with the observed VO^2+^ crossover for N212|Gr and N212|G|N212 membranes, as well as the PCTE+Gr+Nafion membranes in Figure 2.

The diffusive VO^2+^ crossover for N212 ≈0.36 mm h^−1^ and N212|Gr ≈0.23 mm h^−1^ indicates the interfacing of graphene with N212 results in ≈36% decrease in VO^2+^ crossover (Figure 3E). The VO^2+^ crossover corresponds to a VO^2+^ permeability of ≈9.9 × 10^−7^ cm^2^ min^−1^ for N212 and ≈6.4 × 10^−7^ cm^2^ min^−1^ for N212|Gr membranes, respectively (see Table S4, Supporting Information). Interestingly, ≈36% decrease in the VO^2+^ crossover is also observed for N212|Gr|N212 ≈0.15 mm h^−1^ compared to N212||N212 ≈0.23 mm h^−1^ sandwich membranes (Figure 3I). The VO^2+^ crossover corresponds to a VO^2+^ permeability of ≈1.3 × 10^−6^ cm^2^ min^−1^ for N212||N212 and ≈8.3 × 10^−7^ cm^2^ min^−1^ for N212|Gr|N212 membranes, respectively (see Table S4, Supporting Information). The similar reduction in VO^2+^ crossover for N212|Gr and N212|Gr|N212 membranes compared to the respective controls N212 and N212||N212 suggests the reduction is likely due to graphene and percentage area with non‐selective defects or fabrication damages is similar for these membranes despite minor differences in fabrication (see Experimental Section and Figure 3A). Additionally, to observe swelling (due to electrolyte uptake of the polymer, Nafion) induced damages to graphene, we exposed the N212|Gr membrane to 1 M VOSO_4_ + 2 M H_2_SO_4_ and visualized surface morphology of graphene (Figure S11, Supporting Information). We observed no obvious damage in the form of cracks or tears in graphene after electrolyte exposure, suggesting that graphene is able to withstand the swelling‐induced stress/strain (also see discussion of mechanical properties further below).

**Figure 4:**
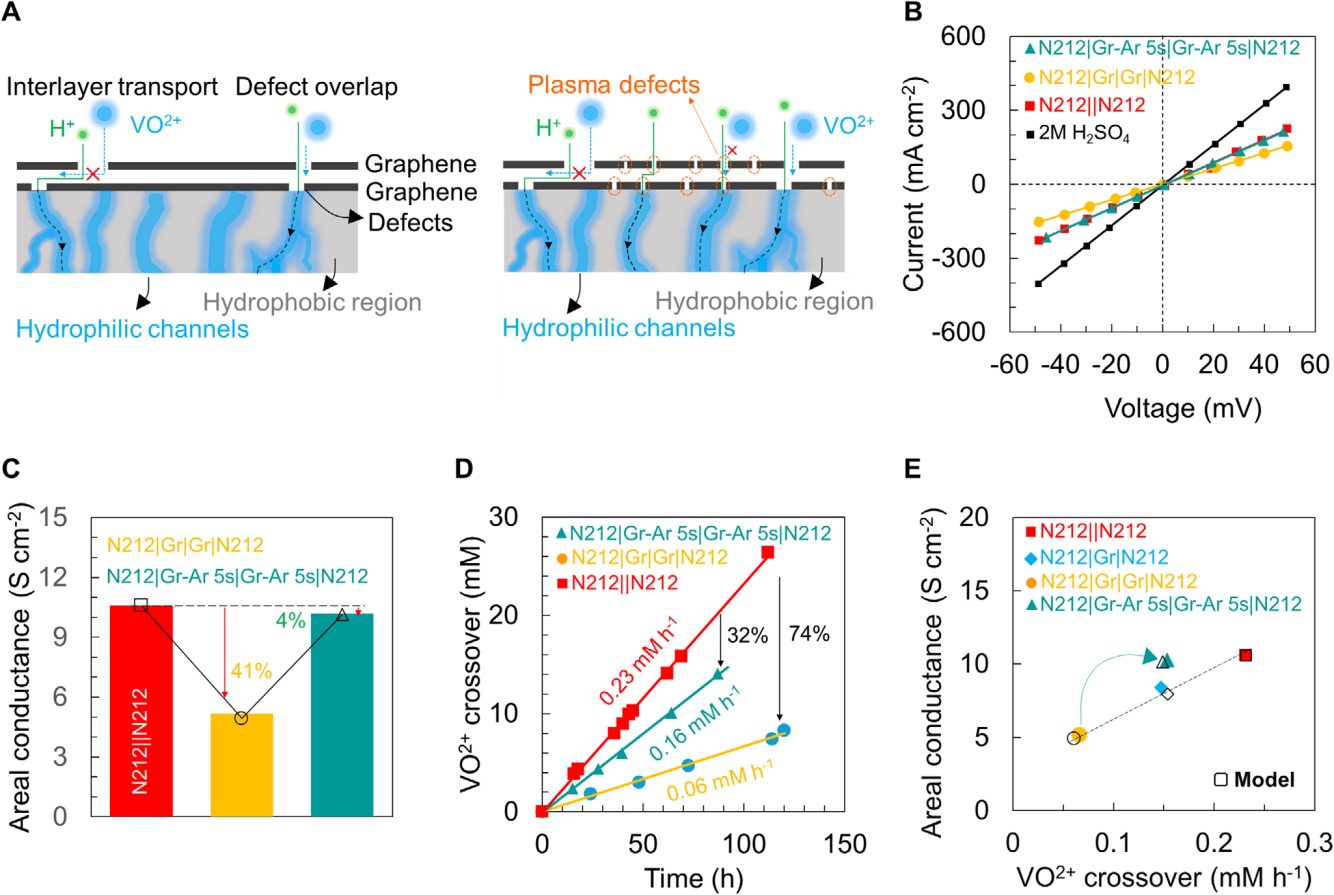
Stochastic isolation and mitigating defects via stacking two layers of monolayer CVD graphene in Nafion 212 sandwich membranes. A) Schematics of transport pathways through two layers of stacked CVD graphene (layer‐by‐layer transfer of two individual monolayers) with and without Ar plasma. The propensity of overlap of tears and defects in two layers is extremely low allowing stochastic isolation of the defects to mitigate VO^2+^ transport while still allowing proton (H^+^) transport. Proton transport pathways include through the graphene lattice, defects/vacancies in the graphene lattice, overlapping tears/large defects in two individual layers of stacked graphene (low probability) as well as interlayer water. VO^2+^ transport is limited to pathways that do not present steric hinderance i.e., larger defects/tears in graphene that allow for VO^2+^ transport. The schematic does not show the top layer of Nafion in the Nafion|Gr|Gr|Nafion sandwich devices. Ar plasma was performed on CVD graphene on Cu foil before transfer (see Experimental Section). B) *I–V* curves for the fabricated membranes in 2 M H_2_SO_4_ and C) corresponding areal conductance computed from B after accounting for solution resistance (see Table S3, Supporting Information). D) VO^2+^ ion crossover as function of time for the fabricated membranes measured using 1 M VOSO_4_ + 2 M H_2_SO_4_ in the feed side and 1 M MgSO_4_ + 2 M H_2_SO_4_ in the permeate side. E) Areal conductance as a function of VO^2+^ ion crossover for the fabricated membranes. Experimental values (filled symbols) show good agreement with computed values from the resistance‐based transport model (open symbols).

Next, we leverage facile stacking of two graphene layers that allows for stochastic isolation of defects due to the low propensity of defects in two separate layers to precisely overlap (see schematic in **Figure** **4**A) and evaluate its potential as a defect mitigation approach for VRFB membranes.^[^ ^52^, ^71^ ^]^ Decrease in areal proton conductance from defect isolation and higher graphene coverage from stacking two layers can potentially be mitigated by introducing proton‐selective defects via exposure to Ar plasma (Figure 4A), as discussed in previous sections. In addition to defect isolation, the interlayer spacing between two stacked individual graphene layers could allow for H^+^ transport while presenting more steric hinderance to the transport of the larger hydrated VO^2+^ ion (Figure 4A).^[^ ^52^ ^]^

Figure 4B show the *I–V* curves for N212||N212 (control), N212|Gr|Gr|N212 and N212|Gr‐Ar 5s|Gr‐Ar 5s|N212 membranes measured in 2 M H_2_SO_4_. The corresponding areal conductance is shown in Figure 4C (after solution resistance correction, also see Table S3, Supporting Information) and VO^2+^ crossover as a function of time in Figure 4D, respectively. The incorporation of two layers of CVD graphene results in areal conductance of ≈5.2 S cm^−2^ with VO^2+^ crossover of ≈0.06 mm h^−1^ for N212|Gr+Gr|N212 membrane, in comparison to areal conductance of ≈10.6 S cm^−2^ and crossover of ≈0.23 mm h^−1^ for N212||N212. The stacking approach results in ≈41% decrease in areal conductance with ≈74% reduction in VO^2+^ crossover. However, 5 s Ar plasma exposure to each of the two layers of graphene (N212|Gr‐Ar 5s+Gr‐Ar 5s|N212) shows areal conductance ≈10.3 S cm^−2^ and crossover ≈0.16 mm h^−1^ representing a mere ≈4% reduction in areal proton conductance but ≈32% reduction in crossover compared to N212||N212 control membranes. Figure 4E shows the areal conductance as a function of VO^2+^ crossover with a clear trade‐off between conductance and crossover. Notably, the 5 s Ar plasma‐treated graphene membrane, N212|Gr‐Ar‐5 s|Gr‐Ar‐5 s|N212 membranes seem to overcome this trade‐off trend and are further corroborated via the resistance‐based transport model (open symbols in Figure 4E). Notably, the plasma treated membrane, N212|Gr‐Ar 5s|Gr‐Ar 5s|N212 exhibits a selectivity of ≈12 × 10^6^ S min cm^−4^ (higher than the N212||N212 ≈8.2 × 10^6^ S min cm^−4^ while retaining the areal conductance ≈10.3 S cm^−2^ (compared to N212||N212 ≈10.6 S cm^−2^) indicating the usefulness of stacking Ar plasma treated two layers of monolayer CVD graphene as a potential route to increase selectivity without compromising areal conductance.

### 2.3 Ultra‐Thin ≈300 nm PBI Layer for Nanoporous CVD Graphene Integration with Nafion 211 for Advanced PEMs for VRFBs

**Figure 5:**
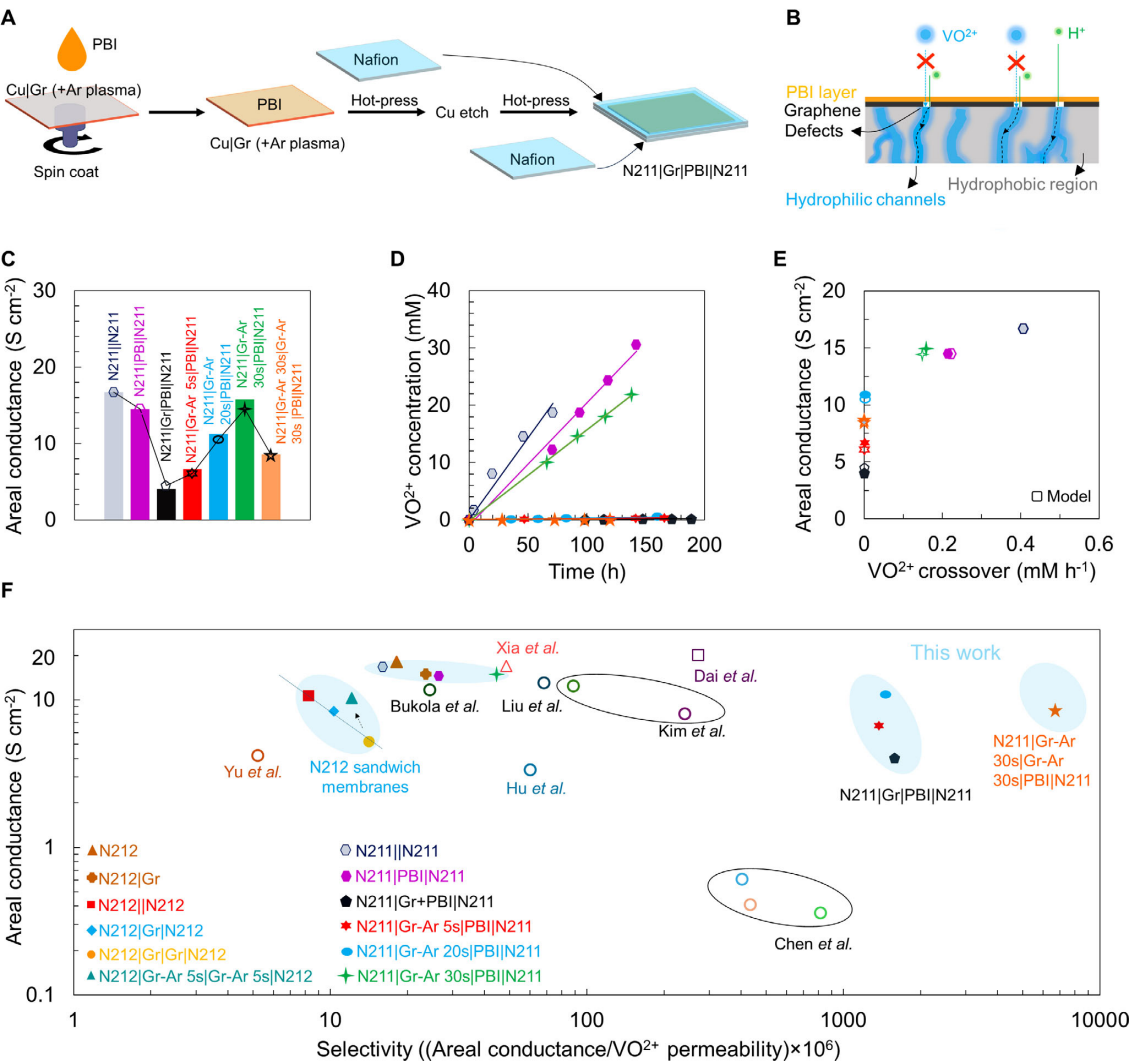
Fabrication of Nafion|Gr|poly‐benzimidazole (PBI)|Nafion sandwich membranes with low VO^2+^ crossover. A) Schematic of fabricating Nafion 211 (≈25 µm thick)|Gr| PBI| Nafion 211 sandwich membranes. B) Schematic of ion transport pathways in Nafion+Gr+PBI+Nafion sandwich membranes (also see Figure S13, Supporting Information). C) Areal proton conductance measured in 2 M H_2_SO_4_ and after correction of solution resistance (see Table S3, Supporting Information). D) Crossover of VO^2+^ ions as function of time measured using 1 M VOSO_4_ + 2 M H_2_SO_4_ in the feed side and 1 M MgSO_4_ +2 M H_2_SO_4_ in the permeate side. E) Areal proton conductance as function of VO^2+^ crossover rate for the fabricated membranes. Filled symbols are experimental measurements and unfilled/open symbols are computed from the resistance‐based transport model. Experimental results show good agreement with computed values. F) Areal proton (H^+^) conductance as a function of membrane selectivity (ratio of areal H^+^ conductance and VO^2+^ permeability, S min cm^−4^). The areal conductance and selectivity values are computed from the data provided in references Yu et al.^[^ ^28^ ^]^ 2016; Chen et al.^[^ ^33^ ^]^ 2017; Kim et al.,^[^ ^26^ ^]^ 2018; Hu et al,^[^ ^23^ ^]^ 2019; Liu et al.^[^ ^32^ ^]^ 2019; Dai et al.^[^ ^24^ ^]^ 2020; Bukola et al.,^[^ ^34^ ^]^ 2021 and Xia et al.,^[^ ^35^ ^]^ 2023.

Although stacking two monolayers of CVD graphene provided valuable insights to increase selectivity, non‐negligible VO^2+^ crossover was observed (Figure 4) and sandwiching between two N212 resulted in ≈100 µm thick membranes compared to conventional N212 ≈50 µm. To minimize crossover further, we use poly‐benzimidazole (PBI) with inherently low VO^2+^ crossover as well as low areal proton conductance for integration of CVD graphene (see schematic in **Figure** **5**A) sandwiched between two Nafion 211 layers resulting in a membrane of comparable thickness to N212 ≈50 µm. We specifically leverage an ultra‐thin layer ≈300 nm of PBI (see Figures S9 and S10, Supporting Information) in combination with graphene with Angstrom‐scale proton‐selective pores to minimize VO^2+^ crossover while allowing high areal proton conductance (see schematic in Figure 5B for transport pathways, alse see Figure S13, Supporting Information).

The measured areal proton conductance (Figure 5C after correction of solution resistance, also see Table S3, Supporting Information) for the synthesized membranes N211||N211 ≈16.7 S cm^−2^, N211|PBI|N211 ≈14.5 S cm^−2^, and N211|Gr|PBI|N211 ≈4.1 S cm^−2^, show ≈74% reduction for the graphene membranes with PBI compared to ≈20% for Nafion integration without the PBI (Figure 4). SEM images of graphene integration with PBI show uniform transfer devoid of the defects or damages seen for standard Nafion integration via hot press indicating the role of improved graphene transfer (see Figure S9, Supporting Information) in the observed reduction in areal conductance. To increase proton conductance, we leverage Ar plasma (as shown in Figures 2 and 4) and observe a systematic increase with increasing plasma exposure time for N211|Gr‐Ar 5 s|PBI|N211 ≈6.7 S cm^−2^, N211|Gr‐Ar‐20s|PBI|N211 ≈10.9 S cm^−2^, and N211|Gr‐Ar‐30s|PBI|N211 is ≈14.9 S cm^−2^, respectively (Figure 5C).

Figure 5D indeed confirms a considerable decrease in VO^2+^ crossover with PBI for the fabricated membranes N211||N211 ≈0.41 mm h^−1^, N211|PBI|N211 ≈0.22 mm h^−1^, N211|Gr|PBI|N211 ≈0.001 mm h^−1^, N211|Gr‐Ar‐5s|PBI|N211 ≈0.001 mm h^−1^, N211|Gr‐Ar‐20s|PBI|N211 ≈0.003 mm h^−1^, and N211|Gr‐Ar‐30s|PBI|N211 ≈0.158 mm h^−1^, respectively. Areal conductance as a function of VO^2+^ crossover rate in Figure 5E confirms these observations and the integration of graphene (with PBI) leads to ≈74% (in conductance) and ≈99% (in VO^2+^ crossover) in comparison to N211||N211 membrane. Ar plasma exposure increases both H^+^ conductance as well as VO^2+^ crossover. However, N211|Gr‐Ar‐30s|PBI|N211 membrane shows ≈11% lower H^+^ conductance and ≈61% lower crossover for VO^2+^ ions in comparison to N211||N211 membrane and N211|Gr‐Ar‐20s|PBI|N211 membranes shows ≈35% lower H^+^ conductance and ≈99% lower crossover for VO^2+^ ions in comparison to N211||N211 membrane. Despite these reductions, the areal conductance remains suitable for practical applications. Such a tradeoff between conductance vs crossover is also observed from the resistance‐based transport modelling (Figure 5E, open symbols, also see Table S2 in Supporting Information). Taken together with Figure S9 (Supporting Information) these observations indicate a pronounced reduction in the large defects for PBI based graphene transfers resulting in superior integration of graphene as well as higher resistance to VO^2+^ crossover from the ≈300 nm PBI in areas of graphene defects/damage, thereby providing transport resistance based sealing in combination with the Angstrom‐scale proton selective pores in CVD graphene.

To further understand the influence of swelling of Nafion with the incorporation of PBI and CVD graphene, we soaked the fabricated membranes in water and electrolyte (1 M VOSO_4_ + 2 M H_2_SO_4_) for 24 h to ensure complete uptake (see Table S5, Supporting Information). As a control, the swelling for Nafion N211||N211 (≈50 µm thickness) membrane was found to be in good agreement with literature for Nafion N212 membrane (≈50 µm thickness).^[^ ^72^ ^]^ The integration of PBI and graphene decreases swelling in both through‐plane and in‐plane directions compared to bare Nafion in water as well as electrolyte (see Table S5, Supporting Information for details). These observations indicate that in‐plane stretching for graphene could be lowered with addition of PBI potentially minimizing damage to graphene via in‐plane stress/strain. However, the decrease in through‐plane swelling with the addition of PBI and graphene in comparison to Nafion (Table S5, Supporting Information) also suggests a potential decrease in proton conductance (or increase in proton resistance) due to less water uptake and found to be in agreement with experimental observations in Figure 5. Cross‐sectional SEM images of composite membranes (see Figure S14, Supporting Information) after proton transport and VO^2+^ crossover measurements for possible delamination of the interface did not indicate any mechanical integrity issues (Figure S15 and Table S6, Supporting Information). The interface observation coupled with the in‐plane swelling suggests adequate mechanical stability of the fabricated membranes (Figure S15 and Table S6, Supporting Information).

Figure 5F shows conductance vs selectivity for all the fabricated membranes and the plot exhibits trend lines informing tradeoffs between conductance and selectivity for the N211 membranes. However, the graphene integrated with PBI and 5, 20, and 30 s of plasma exposure show much higher selectivity while maintaining adequate areal conductance (provided in brackets for corresponding membranes) for VRFB applications: N211||N211 ≈16.1 × 10^6^ S min cm^−4^ (≈16.7 S cm^−2^), N211|PBI|N211 ≈26.5 × 10^6^ S min cm^−4^ (≈14.5 S cm^−2^), N211|Gr|PBI|N211 ≈1729 × 10^6^ S min cm^−4^ (≈4.4 S cm^−2^), N211|Gr‐Ar‐5 s|PBI|N211 ≈1377 × 10^6^ S min cm^−4^ (≈6.7 S cm^−2^), N211|Gr‐Ar‐20 s+PBI|N211 ≈1462 × 10^6^ S min cm^−4^ (≈10.9 S cm^−2^), and N211|Gr‐Ar‐30 s|PBI|N211 ≈44.5 × 10^6^ S min cm^−4^ (≈14.9 S cm^−2^), respectively. Notably, the N211|Gr‐Ar‐20 s|PBI|N211 membrane has ≈121× higher selectivity in comparison to N212|Gr‐Ar‐5 s|Gr‐Ar‐5 s|N212 membrane while maintaining similar areal conductance ≈10.9 S cm^−2^. With exposure of 30 s plasma, selectivity decreases for N211|Gr‐Ar‐30 s|PBI|N211 membrane, but the selectivity is still 3.7× higher than N212|Gr‐Ar‐5 s|Gr‐Ar‐5 s|N212 membrane indicating the incorporation of graphene with PBI as a viable route for fabricating proton selective membranes for VRFBs. In addition to increased selectivity, the N211|Gr‐Ar‐30 s|PBI|N211 membrane also has higher proton conductance ≈1.4× higher in comparison to N212||N212 (proton conductance ≈10.2 S cm^−2^) membrane.

Finally, we leverage the insights from facile stacking for stochastic isolation (Figure 4) in conjunction with integrating graphene with PBI (Figure 5) to fabricate Ar plasma‐treated stacked graphene (stacking individual monolayers of CVD graphene with 30 s of Ar plasma exposure layer by layer) i.e., N211|Gr‐Ar 30 s|Gr‐Ar 30 s|PBI|N211. Such an approach allows us to maximize Angstrom‐scale proton‐selective defects while minimizing fabrication‐induced tears/defects as well as blocking non‐selective defects (Figure 5F). The N211|Gr‐Ar 30 s|Gr‐Ar 30 s|PBI|N211 membrane ≈50 µm in thickness exhibits H^+^ conductance of ≈8.6 S cm^−2^ (Figure 5C, also see Table S3, Supporting Information) while showing a record low VO^2+^ crossover ≈0.0005 mm h^−1^ (Figure 5E and Figure S12, Supporting Information), resulting in selectivity ≈6709 × 10^6^ S min cm^−4^ representing a ≈671× improvement over N212 of comparable thickness. The resistance‐based modelling matches well with these experimental observations and Figure S13 (Supporting Information) provides a simplistic sketch of proton transport pathways at the interface as well as between two stacked layers of nanoporous graphene.^[^ ^73^, ^74^, ^75^, ^76^ ^]^

Finally, comparison of areal conductance vs selectivity for the N211|Gr‐Ar 30 s|Gr‐Ar 30 s|PBI|N211 (Figure 5F) with other literature reports^[^ ^23^, ^24^, ^26^, ^28^, ^32^, ^33^, ^34^, ^35^ ^]^ indeed confirms the notable improvement in the performance.

## 3 Conclusion

In this study, we present a layered proton exchange membrane (PEM) architecture that integrates atomically thin CVD graphene featuring Angstrom‐scale, proton‐selective pores with ultrathin polybenzimidazole (PBI) and commercial Nafion membranes to address the long‐standing trade‐off between proton conductivity and vanadium ion (VO^2^⁺) crossover for VRFBs. Through systematic experimentation and modeling, we demonstrate that large, non‐selective defects—such as cracks and tears in monolayer graphene— dominate crossover and must be isolated or sealed to achieve high selectivity. We show that transferring graphene onto porous supports reveals the role of macroscopic defects, and that plasma‐exposure induced Angstrom‐scale pores can enhance proton transport but must be balanced against increased VO^2^⁺ leakage. Incorporation of a thin (≈300 nm) PBI layer between graphene and Nafion enables improved integration and defect sealing. Stacking two Ar plasma‐treated graphene monolayers further isolates non‐selective defects stochastically and yields a layered membrane (N211|Gr‐Ar 30 s|Gr‐Ar 30 s|PBI|N211) with record‐high selectivity of ≈6709 × 10⁶ S min cm^−^⁴ and areal proton conductance >8 S cm^−^^2^—an over 670× enhancement in selectivity relative to Nafion 212. These findings establish a new design paradigm for high‐performance PEMs by combining atomic‐scale defect engineering in 2D materials with strategic layering and stacking. This scalable approach offers a generalizable platform for developing next‐generation membranes not only for VRFBs, but also for a wide range of energy, electrochemical, and molecular separation applications.

## 4 Experimental Section

### Materials Section

All the chemicals and materials used in this study are described below. Nafion (N211, N212 procured from Ion Power), Poly‐benzimidazole (Celazole), PMMA (Acros Organics, MW 35000, 2 wt.% in anisole). Organic solvents such as acetone and isopropyl alcohol (IPA) were procured from Fisher Chemical. Chemical etch test of graphene was done using 0.1 M FeCl_3_ (EMD Millipore Corporation, >98.0% assay). Composite membranes were fabricated using a 10‐ton hydraulic press with temperature control (Dabpress). For graphene transfers, underlying Cu removal was performed in ammonium persulfate (0.2 M, Thermo Scientific, ACS reagent 98+%). The UV–vis spectra were collected using UV–vis spectroscopy (Agilent Cary 60). Graphene synthesized via atmospheric pressure chemical vapor deposition (CVD) on Cu foil was sourced from General Graphene Corporation LLC.

### Etch Tests for Defect Analysis of CVD Graphene on Cu Foil

Etch tests for defect analysis of the CVD graphene on Cu foil,^[^ ^57^, ^69^ ^]^ was performed by placing a 5 µL drop of 0.1 M FeCl_3_ in de‐ionized (DI) water for 5 s, followed by rinsing in DI water and drying at ambient conditions. Finally, the etch pits formed were imaged using a scanning electron microscope (SEM, Zeiss Merlin) at an acceleration voltage of ≈2 kV.

### Raman Spectroscopy

Raman spectroscopy was performed on CVD graphene transferred to 300 nm SiO_2_/Si wafer using a sacrificial PMMA carrier layer (removed using acetone and IPA after transfer) using a confocal Raman microscope (Thermo Scientific DXR) with a 532 nm wavelength laser and a grating of 900 lines per mm. Raman spectra were averaged over 30 scans with an exposure of 20 s under an excitation of 2 mW power.

### Graphene Transfer to Polycarbonate Track Etched (PCTE) Supports

Graphene transfer to polycarbonate track‐etched (PCTE, Sterlitech, 10% membrane porosity and pore diameter ≈200 nm) was performed via isopropanol (IPA) assisted hot lamination process as reported previously.^[^ ^64^, ^66^ ^]^ CVD graphene on Cu was placed on PCTE supports and a drop of IPA was added to the interface to facilitate conformal contact as the Cu/graphene/PCTE stack was sandwiched between Teflon pieces for lamination at 135 °C using an office laminator (Trulam, TL‐320). The Cu foil was etched by floating the Cu/Graphene/PCTE stack on ≈0.2 M ammonium persulfate (APS) solution and the resulting CVD graphene/PCTE stack was floated on DI water twice (≈10 min each), rinsed in ethanol, and dried in ambient.

### Graphene Transfer to TEM Grids

A polymer‐free approach was utilized to transfer CVD graphene (both pristine as well as graphene exposed to Ar plasma) to Holey Carbon on 300 Mesh Au TEM grids (Electron Microscopy Sciences).^[^ ^77^, ^78^, ^79^ ^]^ First, the backside of as grown CVD graphene was removed via pre‐etch in 0.2 M APS followed by floating on DI water. Next, the TEM grid was placed on CVD graphene (on Cu foil) and a drop of IPA was added and was allowed to evaporate and dry facilitating adhesion. Finally, the Cu foil was etched by floating the TEM Grid + CVD Graphene on Cu stack on 0.2 M APS and TEM Grid + CVD graphene was floated on DI water to get rid of residual APS. Finally, the TEM grid with graphene was rinsed in ethanol and dried in air at ambient.

### STM Imaging of CVD Graphene on Cu Foil

STM images of CVD graphene on Cu foil were obtained with an Omicron variable temperature scanning tunneling microscope (VT‐STM) at room temperature in the Center for Nanophase Materials Sciences at Oak Ridge National Laboratory.^[^ ^52^ ^]^ Before acquiring the images, the CVD graphene on Cu samples were annealed under ultra‐high vacuum at 500 °C for 1 h.

### STEM Imaging of CVD Graphene Transferred onto TEM Grids

ADF‐STEM images were acquired using a JEOL ARM200F NEOARM equipped with a CEOS ASCOR corrector operated at an accelerated voltage of 80 kV with an aperture of 40 microns and a convergence semi‐angle of 27 milliradians. Prior to imaging, graphene sample was annealed in vacuum at ≈200 °C for 12 h to minimize contamination.

### Fabrication of Nafion Filled PCTE and PCTE|Gr Membranes

Nafion was filled in the pores by soaking the pre‐wetted (with ethanol) porous PCTE (with or without graphene) membrane in 5 wt.% Nafion solution for 30 min under mild vacuum, followed by ambient drying. Next, a second coating of Nafion was performed under identical conditions, followed by drying before assembling them for the ionic conductance measurements.

### Interfacial Polymerization

Tears, damages, and large defects in CVD graphene transferred to PCTE were sealed via interfacial polymerization using branched polyethyleneimine (PEI, MW 600 Da) of ≈1% (w/v) in the aqueous phase i.e., 0.1 g of PEI was dissolved in 10 mL of water and ≈0.1% (w/v) cyanuric chloride (CC) in hexane for ≈1 h.^[^ ^80^, ^81^ ^]^ The membrane was rinsed in hexane, followed by ethanol. Considering the radius, *R_min_* of the PEI molecules ≈1.12 nm, defects larger than the *R_min_* would be sealed via interfacial polymerization.^[^ ^66^, ^67^ ^]^ Note interfacial polymerization was done after pore creation, to avoid any damage to the IP sealed plugs from plasma exposure.

### Diffusion and Pressure‐Driven Transport

Pressure‐driven ethanol and diffusion‐driven solute transport measurements on CVD graphene transferred to PCTE supports were performed using prior approaches.^[^ ^50^, ^57^, ^58^, ^64^, ^66^, ^67^, ^68^ ^]^ A modified diffusion cell (PermeGear, Inc., 5 mm orifice) with a syringe stem was used for pressure‐driven ethanol transport by inducing a hydrostatic head between both half cells while recording the change in the volume (Δ*V*) over time. Permeance (*P)* was calculated using *P* = (Δ*V*/Δ*P*)/(Δ*t* × *A*_effective_), where Δ*P* was the pressure difference across the membrane computed from height difference of ethanol between two sides, Δ*t* was the time interval for measuring the change in volume Δ*V* and *A*_effective_ was the effective membrane area. Normalized flux was computed by taking the ratio of permeance for graphene|PCTE with respect to bare PCTE supports.

Diffusion‐driven transport measurements were performed after the pressure‐driven ethanol transport. Initially, the membranes were mounted into the cells and wetted with ethanol and rinsed five times with DI water, before measurements for KCl (Fisher Chemical, 7447‐40‐7, 74.55 Da), and Vitamin B12 (B12, Sigma–Aldrich, 68‐19‐9, 1355 Da) were performed. For KCl (hydrated diameter of K^+^ and Cl^−^ ≈0.66 nm), 0.5 M of KCl solution was filled into the feed side, and DI water was filled into the permeate side and increase in permeate side conductivity was recorded using a conductivity meter (Mettler Toledo SevenCompact S230).^[^ ^64^, ^65^ ^]^ For B12 (molecular diameter ≈1–1.5 nm), the feed side was filled with 1 mm B12 in 0.5 M KCl and the permeate side with 0.5 M KCl. The increase in B12 concentration (corresponding to the peak intensity at 360 nm wavelength) on the permeate side was measured using UV–vis spectrophotometer (Agilent Cary 60). Permeance (*P*) was computed using *P* = (V × dC/dt)/(ΔC × A_effective_), where V was the volume of diffusion cell (7 mL), dC/dt was the slope of concentration change in the permeate side, ΔC was concentration difference between feed side and permeate side, and A_effective_ was the effective area of membranes (accounting for 10% porosity of PCTE). Normalized flux for pressure and diffusive transports was computed using the ratio of permeance of graphene|PCTE to bare PCTE support.

### Fabrication of Nafion|Graphene (Gr), Nafion|Gr|Nafion, and Nafion|Gr|Gr|Nafion Sandwich Membranes

As received Nafion (N212, 50 µm thick or N211, 25 µm thick) was treated in 0.5 M H_2_SO_4_ at 80 °C for 1 h followed by a rinsing in water at 80 °C for 1 h. Next, the N212 membranes were soaked in 0.1 M H_2_SO_4_ for 48 h to ensure complete conversion to H^+^ form and subsequently rinsed in water and dried in ambient before graphene transfer. CVD graphene on Cu foil was hot‐pressed on N212 at 145 °C for 3 min under ≈625 psi as per prior reports.^[^ ^52^, ^57^ ^]^ Next, the Cu was etched in 0.2 M APS followed by 2–3 DI water rinses and drying in ambient to form N212|Gr membranes. To form the N212|Gr|N212 sandwich membrane another layer of N212 was hot‐pressed onto N212|Gr at 145 °C for 3 min under ≈625 psi on the N212|Gr membrane. To fabricate a two‐layer (layer by layer) Nafion|Gr|Gr|Nafion membrane, two individual Nafion|Gr layers (prepared via same approach as mentioned above) were hot‐pressed together at 145 °C for 3 min under ≈625 psi with graphene facing toward each other (also see Figure S16, Supporting Information). Floating in 0.1 M HCl after graphene transfer to Nafion was avoided since the membranes were soaked and measured in H_2_SO_4_.^[^ ^82^ ^]^

### Proton Selective Nanopore Formation in CVD Graphene via Ar Plasma

Argon (Ar) plasma at desired power (medium power ≈11 W and high power ≈37 W), pressure ≈500 mTorr, time ≈1–120 s using a Plasma Cleaner (Harrick Plasma) was used to introduce proton‐selective pores in the CVD graphene while on Cu foil for composite membranes with Nafion (to avoid plasma damage to Nafion). Ar plasma was performed after CVD graphene transferred via IPA‐assisted hot‐lamination to PCTE support or sacrificial PMMA‐assisted transfer to 300 nm SiO_2_|Si for Raman spectroscopy. For the PMMA sacrificial layer‐assisted CVD graphene transfer, PMMA was dissolved in acetone after graphene transfer, followed by rinsing in IPA and drying at ambient before exposure to plasma for nanopore/defect creation.

### Fabrication of Nafion|Gr|PBI|Nafion Membranes with Monolayer and Two‐Layer Graphene

Poly‐benzimidazole (PBI, 5 wt.% in dimethylacetamide, DMAc), was spin‐coated (500 µL of 5 wt.% PBI solution ≈1 min, ≈1000 rpm) onto CVD graphene on Cu foil. The spin‐coated Cu|Gr+PBI was dried at ambient for 30 min, followed by baking at 80 °C for ≈2–3 h to remove the residual solvent before Nafion 211 (≈25 µm thick) was hot‐pressed using the process described above. The thickness of the spin‐coated PBI layer was measured using the KLA Tencor P‐7 Profilometer on a spin‐coated PBI (with graphene membranes) transferred to SiO_2_/Si wafer, see Figure S10 (Supporting Information). The measured thickness for spin coated PBI layer was ≈300 nm.

To fabricate a two‐layer (layer by layer) CVD graphene membrane with 30 s of Ar plasma exposure for each layer, first, a Cu+Gr‐Ar 30 s+PBI layer was prepared via spin coating PBI on Cu+Gr‐Ar 30, as stated above. Next, the Cu layer was etched away, the resulting Cu+Gr‐Ar 30 s+PBI layer was washed in water and then it was scooped on second layer of graphene (Cu+Gr‐Ar 30 s) to make a bilayer membrane. Next, the resulting stack was dried and from here onward following the fabrication steps of single‐layer graphene (with PBI) i.e., hot‐press, removal of Cu, and water wash, a stacked bilayer graphene membrane was obtained, labeled as “N211|Gr‐Ar 30 s|Gr‐Ar 30 s|PBI|N211.”

### Ionic Conductance Measurements

A custom‐built H‐cell with Luggin capillaries (see schematic in Figure S5, Supporting Information) was used for acquiring the *I–V* curves.^[^ ^52^, ^57^ ^]^ Ionic conductance was computed from *I–V* curves, measured in 2 M H_2_SO_4_ using a four‐electrode configuration. Pt wire electrodes work as working and counter electrodes, whereas Ag/AgCl electrodes as reference and working sense electrodes.

The graphene resistance was estimated following a series resistance model and shown below as an example for the Nafion sandwich membrane. Total membrane resistance from *I–V* corresponding to N212|Gr|N212 is given as,

(1) $$Total\;resistance\;\left(R_{N212\left|Gr\right|N212+solution}\right)=2\times R_{S}+2\times R_{N212}+R_{Gr}$$

where, 2 × *R_S_* is the total solution resistance of the system. The “N212|Gr|N212” resistance after solution correction can be written as,

(2) $$R_{N212\left|Gr\right|N212}=R_{N212\left|Gr\right|N212+solution}-\left(2\times R_{S}\right)$$

Similarly, “Gr” resistance after N212 and solution resistance correction can be written as,

(3) $$R_{Gr}=R_{N212\left|Gr\right|N212+R_{S}}-\left(2\times R_{S}+2\times R_{N212}\right)$$

Areal conductance (inverse of resistance) values for the sandwich membranes (with and without graphene) were reported after correction for the solution resistance (see Tables S1 and S3, Supporting Information).

### VO^2+^ Ion Crossover Measurements

VO^2+^ ion crossover measurements were performed on the same membranes after *I–V* measurements by assembling them in a side‐by‐side diffusion‐cell. Two compartments of the cell were filled with isotonic solutions (to eliminate the driving force arising from osmotic pressure) of 1 M VOSO_4_ + 2 M H_2_SO_4_ (feed side) and 1 M MgSO_4_ + 2 M H_2_SO_4_ (permeate side). A stirring rate of 1500 rpm was used throughout the crossover experiment to minimize concentration polarization. Aliquots were pipetted from the permeate side, and corresponding UV–vis spectrum was recorded at different time intervals to obtain concentrations using a calibration curve. The concentration vs. time plot provides the rate of VO^2+^ ions crossover.

Permeability (*P*) of the VO^2+^ ions is estimated using,

(4) $$P=\left(\frac{V\;\times \;l}{A\;\times \;\left(C_{0}-C_{m}\right)}\right)\times \frac{dC_{m}}{dt}$$

where *V* is the volume of the reservoir, *l* is the membrane thickness (50–100 µm), *A* is the membrane area (≈0.2 cm^2^), *C_0_* is the VO^2+^ ions concentration in feed side (1 M) and *C_m_* is the VO^2+^ ions concentration in permeate side. Selectivity of the membrane was calculated as a ratio of areal proton conductance and permeability of VO^2+^ ions.

### Chemical Stability and Mechanical Strength

Chemical stability of Nafion, PBI, and graphene membranes was measured by soaking the membranes in water and 1 M VOSO_4_ + 2 M H_2_SO_4_ for at least 24 h and measuring the corresponding changes in the thickness and area of the membrane. Thickness measurements were performed at multiple locations across the membranes and the corresponding analysis for in‐plane swelling (membrane area) was performed using ImageJ. Mechanical property measurements on the membranes (0.6 × 2 cm) were conducted on an Instron 5944 mechanical testing system with a tensile speed of 5 mm min^−1^.

### Resistance‐Based Transport Model

The three composite graphene membrane designs investigated in this study all provide reduced VO^2+^ crossover with high H^+^ transport through the intrinsic selectivity of graphene. Furthermore, they all achieve improved proton conductance and selectivity through exposure of graphene to Ar plasma. To corroborate the proposed transport mechanisms through these membranes and understand differences in performance between the designs, an analytical transport model was developed. Values quantifying graphene porosity were selected for each design to match measured conductance and crossover data to verify that the proposed transport pathways can explain the results. The graphene porosity parameters provide insight into how fabrication differences between the designs affect the graphene active layer in the final membrane structure and suggest areas for future performance gains.

Figure S8 (Supporting Information) presents the equivalent transport resistance models for the different membrane structures. In all cases, ions were conducted through the support layer on one side, then through the porous graphene layer, and finally through the support layer on the other side. The support layers consist of PCTE pores, Nafion, PBI, and combinations of these in different designs. Since the proton conductance of porous graphene was orders of magnitude higher than that through pristine graphene, the model only incorporates transport through pores.^[^ ^52^, ^57^ ^]^ These pores include large (≥50 nm) tears, smaller defects (≤50 nm), and intentionally created selective pores produced by argon plasma exposure. In single‐layer graphene composite membranes, ions can pass from one support to the other through any of these three parallel pathways (Figure S8A,B,D, Supporting Information).

For the single‐layer graphene membrane designs, combining resistances in Figure S8A,B,D (Supporting Information) gives the overall membrane resistance as,

(5) $$R_{1-layer}=R_{support}+{\left[aR_{tear}^{-1}+\left(1-a\right)R_{defect}^{-1}+\left(1-a\right)R_{selective}^{-1}\right]}^{-1}$$

where *R_tear_*, *R_defect_*, *R_selective_* are the transport resistances through tears, defects, and selective pores, respectively, *R_support_* is the resistance of the support layer, and *a* is the fraction of graphene area occupied by tears. The support resistance was given by *R_support_* = *R_PCTE_* + *R_Nafion_* for the PCTE+Gr+Nafion membrane, *R_support_* = 2*R_Nafion_* for the Nafion|Gr|Nafion membrane, and *R_support_* = 2*R_Nafion_* + *R_PBI_* for the Nafion|Gr+PBI|Nafion membrane. Here, *R_PCTE_*, *R_Nafion_*, and *R_PBI_* are the resistance through the PCTE, Nafion, and PBI layers, respectively. The measured support resistance for each membrane type was used in the model calculations. However, the conductivity of H^+^ and diffusivity of VO^2+^ through the two Nafion types were extracted from measured Nafion resistances using a model for conduction/diffusion through a thin layer,

(6) $$R_{Nafion}A=\frac{L}{k}$$

where *L* is the Nafion thickness, *A* is the membrane area, and *k* is the H^+^ conductivity or VO^2+^ diffusivity for transport by conductance and crossover, respectively.

Since the tear diameter was much larger than the graphene layer thickness, the resistance of tears was modeled as conduction/diffusion through a circular opening in an infinitesimal thickness layer that was otherwise impermeable,^[^ ^83^ ^]^

(7) $$R_{tear}A=\frac{\pi D_{tear}}{4k}$$

where *D_tear_* is the tear diameter. Although tears will have a distribution of sizes because they were non‐selective, the leakage they cause was captured with a single diameter (*D_tear_*) and fractional area coverage (*a*). Since the purpose here was to verify that the proposed transport pathways were a feasible explanation for the measurements, the tears were not modeled in more detail. A value of *D_tear_* = 200 nm was used in the model to match transport measurements.

Smaller defects and selective pores can have pore diameters comparable to the thickness of the graphene layer, which can affect transport rates. The resistance of one of these pores was modeled as conductance through a circular hole in a finite‐thickness layer,^[^ ^84^ ^]^

(8) $$R_{pore}=\frac{4t}{\pi k{\left(D-D_{ion}\right)}^{2}}+\frac{1}{k\left(D-D_{ion}\right)}$$

where *t* = 0.68 nm was the graphene thickness, *D_ion_* is the ion diameter, taken as 8.18 Å for VO^2+^ and zero for H^+^ since protons can conduct through the pristine graphene lattice, and *D* is the pore diameter.^[^ ^5^ ^]^ This equation applies for *D* > *D_ion_*, whereas the transport rate was set to zero for pores smaller than the ion diameter.

Both defects and selective pores had a distribution of sizes that presented parallel transport pathways through the membrane. Exponential probability distributions were used to model both the defect and selective pore populations as,

(9) $$p_{defect}\left(D\right)=\frac{1}{{\bar{D}}_{defect}}e^{-D/{\bar{D}}_{defect}}$$

and

(10) $$p_{selective}\left(D\right)=\frac{1}{{\bar{D}}_{selective}}e^{-D/{\bar{D}}_{selective}}$$

where ${\bar{D}}_{defect}$ and ${\bar{D}}_{selective}$ are the average diameters of defects and selective pores, respectively. Combining resistances in parallel, the resistances due to areas with defects and selective pores are calculated as,

(11) $${\left(R_{defect}A\right)}^{-1}=n_{defect}\int_{D_{ion}}^{\infty }\frac{p_{defect}}{R_{pore}}dD$$

and

(12) $${\left(R_{selective}A\right)}^{-1}=n_{selective}\int_{D_{ion}}^{\infty }\frac{p_{selective}}{R_{pore}}dD$$

where *n_defect_* and *n_selective_* are the area densities of defects and selective pores in regions not occupied by tears.

Interfacial polymerization covers most pores larger than a threshold size. In the model, the interfacial polymerization was approximated as completely sealing all tears as well as defects and selective pores larger than a threshold size of 2.2 nm, selected to match model conductance and crossover measurements. The upper limit of integration was changed to this value in the integrals in Equations (11) and (12) to calculate resistances through membranes after interfacial polymerization, and the tear resistance was made infinite for these membranes.

Stacking two graphene layers can reduce leakage by covering pores in one layer with pristine graphene in the other layer. However, the reduction of both H^+^ conductance and VO^2+^ crossover for a two‐layer graphene membrane on Nafion compared to a one‐layer membrane was similar to the reduction for a one‐layer membrane compared to bare Nafion (Figure 4E). Perfect sealing of pores in one layer by pristine graphene in another layer would result in a greater reduction when a second layer is added, since transport could only occur through the much smaller area where pores in both layers align. The lower measured reduction suggests significant transport between graphene layers connecting laterally offset pores in two graphene layers. The interlayer transport resistance was expected to prevent transport between distant pores in the two layers, but this pathway will have a large effect for nearby pores.

To account for this, a model was used that neglects interlayer transport resistance over short distances relative to the pore size, and neglects transport over large distances between pores (Figure S8C,E, Supporting Information). It was previously found that this model, without selective pores, could explain the measured intrinsic ion conductance of two‐layer graphene membranes.^[^ ^52^ ^]^ Ions that pass through a tear in one layer can either reach a nearby tear in the second layer, or conduct through the smaller defects or selective pores in that layer (two left most branches in Figure S8C,E, Supporting Information). Similarly, ions that pass through defects or selective pores in one layer can reach nearby tears in the second layer, or smaller defects and selective pores in that layer (two right most branches in Figure S8C,E, Supporting Information).

Here, selective pores and defects were grouped together because they have similar sizes and will be scattered together over areas not occupied by tears. Their combined resistance is:

(13) $$R_{defect+selective}={\left(R_{defect}^{-1}+R_{selective}^{-1}\right)}^{-1}$$

Accounting for the fraction of area over which tears in one layer act in series with tears in the other layer, defects and selective pores in one layer act in series with defects and selective pores in the other layer, and tears in one layer act in series with defects and selective pores in the other layer, the total resistance of a two‐layer graphene composite membrane is calculated as,

(14) $$\begin{array}{ccc} & & R_{2-layer}=R_{\sup port}\\ & & +{\left[\frac{a^{2}}{2R_{tear}}+\frac{2a\left(1-a\right)}{R_{tear}+R_{defect+selective}}+\frac{{\left(1-a\right)}^{2}}{2R_{defect+selective}}\right]}^{-1} \end{array}$$

Model parameters were set to match the measured conductance and crossover for each type of composite membrane. Model parameters are provided in Table S2 (Supporting Information) and the results were compared with the experimental measurements in Figures 2E–G; 3C,E, and 5C,E. The model was able to follow the changes in conductivity, crossover, and selectivity with membrane structure demonstrating that the proposed transport mechanisms can explain the experimental results.

It is important to note that the model parameters are all affected by the membrane fabrication process and thus vary between composite membrane types. The graphene transfer procedure had a significant effect on tears and defect sizes and density. Using a hot press vs polymer supported transfer method, and which substrate material was used, will affect the level of material defects in the final composite membrane. Similarly, the electrical properties of the substrate beneath graphene during plasma exposure could affect the resulting pore density and size distribution. Whether graphene was transferred before or after plasma exposure, and the transfer process used, can affect the selective pore distribution of the final composite membrane.

Matching the model to measurements suggests that the PBI supported transfer, or the presence of PBI on graphene, results in lower tear density and smaller effective defect and selective pore size in comparison to the other membrane designs. The Nafion|Gr|PBI|Nafion membranes show increase of conductance with minimal loss of selectivity with 5 and 20 s Ar plasma exposure. This results in similar model values for defect and selective pore diameters, which may be an indication that the graphene transfer process following plasma exposure influences the final size of pores in the membrane. When the plasma exposure was further increased to 30 s, selectivity drops to near the value measured without graphene present. The graphene was nearly destroyed by this longer plasma exposure, reflected in the high model value of selective pore density. It is with units noteworthy that to match measured conductance and crossover data, different values of selective pore density and average pore diameter for PBI‐supported two‐layer graphene membranes were used in the model than for PBI‐supported single‐layer graphene (Table S2, Supporting Information). In particular, the measurements show a strong reduction in VO^2+^ crossover with the addition of the second layer of graphene compared to the first, in contrast to the Nafion support results without PBI, which showed a linear decrease with number of layers (Figure 4E). These results indicate that the PBI membrane transfer procedure provides tighter contact between graphene layers compared to the Nafion membrane transfer, reducing interlayer ion transport, with greater reduction for the larger VO^2+^ ions. However, the results can also be explained by sample‐to‐sample variability in the Ar plasma process. This was confirmed by the good match between measurements and model values for N211|Gr+A5 5 s| Gr+A5 5 s|PBI|N211 after adjusting the average pore diameter and density from the single‐layer graphene values.

## Conflict of Interest

P.R.K. declares stake in a company aimed at commercializing 2D materials.

## Author Contributions

P.R.K. conceived and supervised the project. P.C. #1 fabricated membranes and performed electrical and vanadium diffusion driven transport measurements. P.C. #2 performed transfer to PCTE and associated diffusion‐driven (with KCl) and pressure‐driven (with ethanol) transport experiments. S.M.H. and A.L. performed STM imaging at ORNL. M.C. and J.W. performed STEM imaging. M.S.H. performed resistance‐based transport modelling and wrote the corresponding sections of the manuscript. P.C. #1 and P.R.K. wrote the paper with discussions from all authors.

## Supporting information



Supporting Information

## Data Availability

The data that support the findings of this study are available in the supplementary material of this article.
