# Supplementary material for: Proton Selective Nanoporous Atomically Thin Graphene Membranes for Vanadium Redox Flow Batteries
Source: Adv Mater. 2025 Nov 12;38(5):e10609. doi: 10.1002/adma.202510609 (PMC12822541; doi:10.1002/adma.202510609)
Supplement: Supplementary file 1 — Supporting Information [file ADMA-38-e10609-s001.pdf]

ADVANCED MATERIALS

Supporting Information

for *Adv. Mater.*, DOI 10.1002/adma.202510609

Proton Selective Nanoporous Atomically Thin Graphene Membranes for Vanadium Redox Flow Batteries

*Pavan Chaturvedi, Peifu Cheng, Saban M. Hus, Matthew Coupin, An-Ping Li, Jamie Warner, Michael S.H. Boutilier and Piran R. Kidambi**

Supporting Information

Proton Selective Nanoporous Atomically Thin Graphene Membranes for Vanadium Redox Flow Batteries

*Pavan Chaturvedi,[1] Peifu Cheng,[1] Saban M. Hus,[2] Matthew Coupin,[3] An-Ping Li,[2] Jamie Warner,[3] Michael Boutilier,[4] Piran R. Kidambi[3,5,]**

[1] *Department of Chemical and Biomolecular Engineering, Vanderbilt University, Nashville, Tennessee 37212, United States.*

[2] *Center for Nanophase Materials Sciences, Oak Ridge National Laboratory, Oak Ridge, Tennessee, 37831, United States.*

[3] *Walker Department of Mechanical Engineering, University of Texas at Austin, Austin, Texas 78712-1591, United States.*

[4] *Department of Chemical and Biochemical Engineering, Western University, London, Ontario, Canada*

[5] *Department of Mechanical and Aerospace Engineering, University of Florida, Gainesville, Florida, United States.*

*E-mail: p.kidambi@ufl.edu

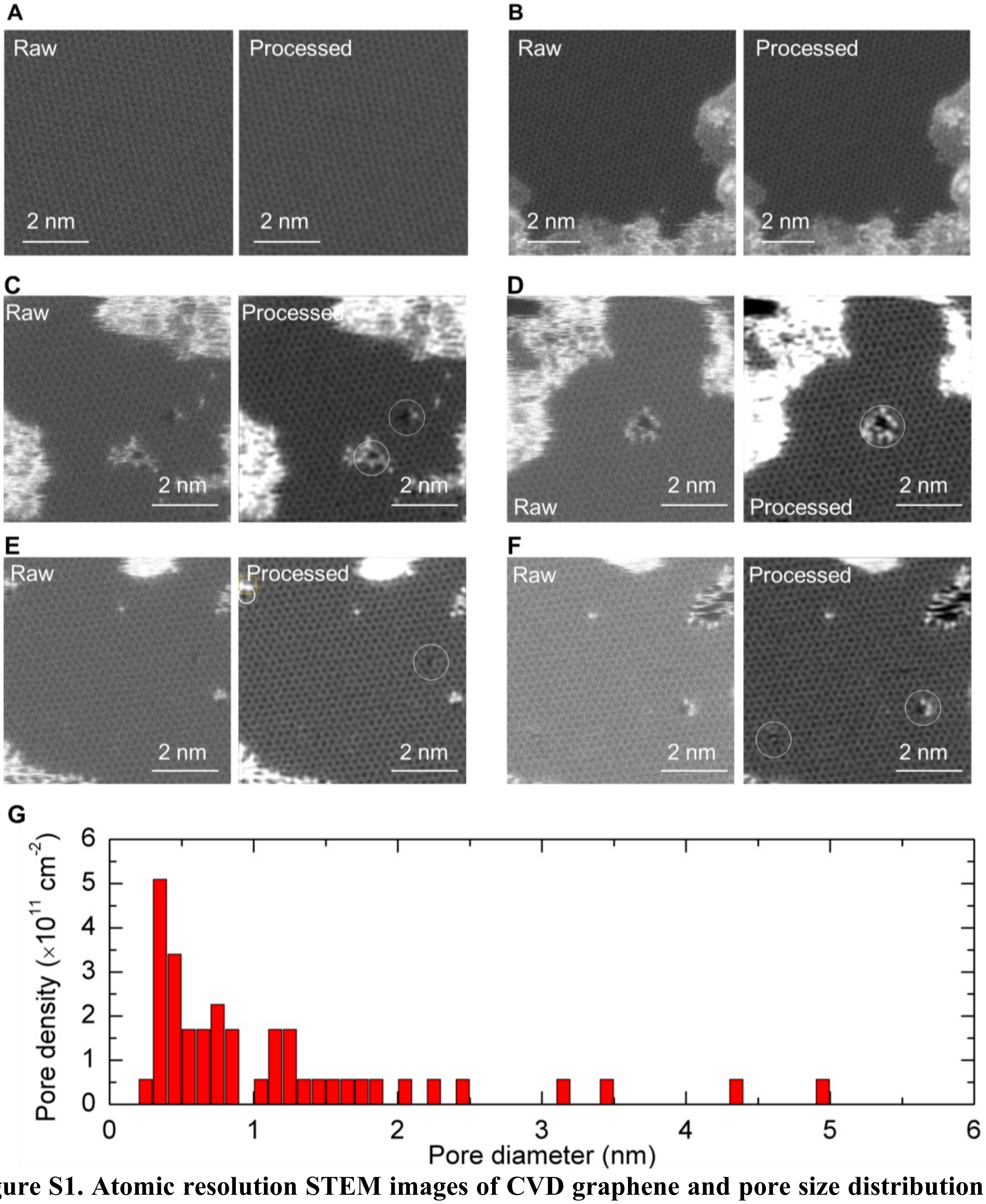

Figure S1. Atomic resolution STEM images of CVD graphene and pore size distribution of defects in CVD graphene after exposure to 30s of Ar plasma extracted from STEM images. Representative STEM images of A, B) pristine (un-treated) CVD graphene lattice, and C-F) graphene lattice after 30s of Ar plasma exposure on CVD graphene on Cu and G) pore size distribution of the corresponding defects. The acquired images are labelled as "Raw" and after FFT correction images are labelled as "Processed". FFT correction was performed using ImageJ software. Defects are marked with white circles in the processed images. Adsorbed contaminants could cover defects in the graphene lattice and hence STM was also performed on graphene on Cu foil after Ar plasma treatment (see Figure 1B,F, and S2).

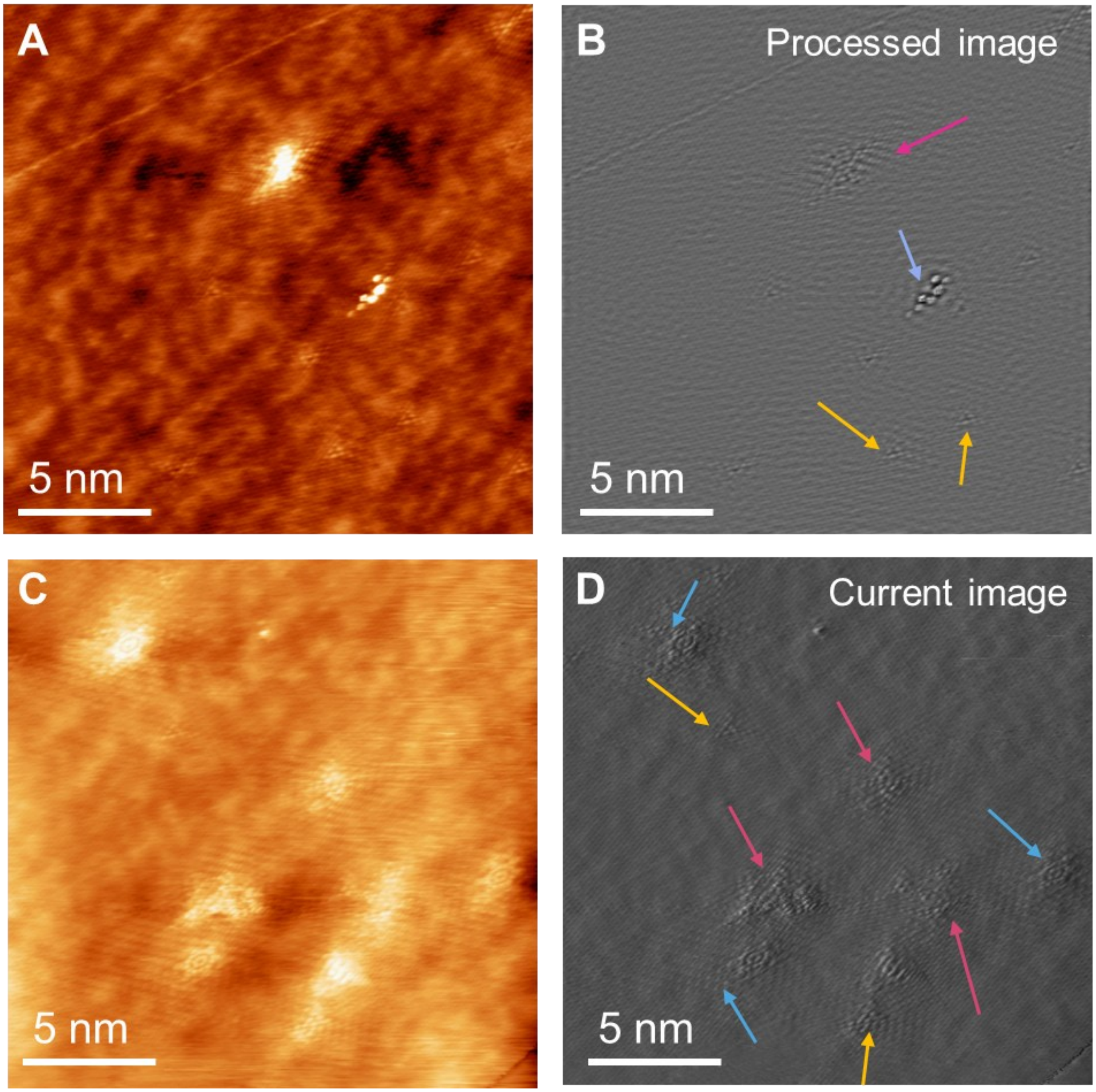

Figure S2. STM images of CVD graphene on Cu foil after exposure of 20s of Ar plasma.
A) and C) Surface topography of CVD graphene on Cu foil after 20s of Ar plasma treatment and their corresponding processed imaged in B) and D). Processed images showing the presence of defects more clearly. Defects are indicated by arrows - point defects (yellow), multi-vacancy defects (purple) and other defects (blue) are observed.

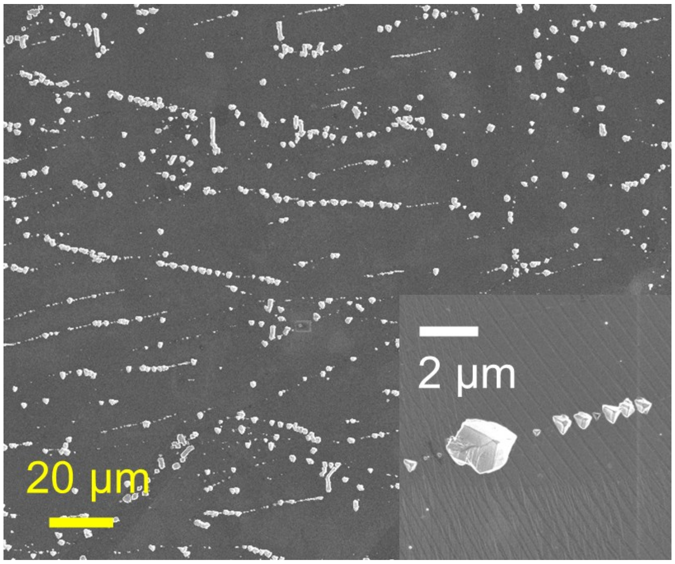

Figure S3. SEM image of CVD graphene on Cu foil after acid etch test. SEM images of as-synthesized CVD graphene on Cu after acid etch test with 0.1M $FeCl_3$ for 5s. The bright spots are the etch pits in Cu formed underneath defects in CVD graphene.

For the defect analysis, etch test[1–3] reveals the defects in CVD graphene by etching pits underneath the Cu that appear as bright dots in SEM images. It was hypothesized that a single defect leads to a single etch pit nucleation and presence of multiple defects nearby (likely along the wrinkles) may lead to coalescing of etch pits as seen in the form of line patterns.[1]

The etched area is ~ 6 % of the total area and considering a single etch pit corresponds to a single defect will result in an etch pit defect density of ~ 2.6×10^6 defects/cm^2 (total etch counts ~ 1501, area ~ 300.0×195.3 μm^2). The defect density is in the range of prior literature[2] defect density of ~ 7.1×10^6 defects/cm^2. Differences could originate from coalescence of multiple defects resulting in a lower defect density.

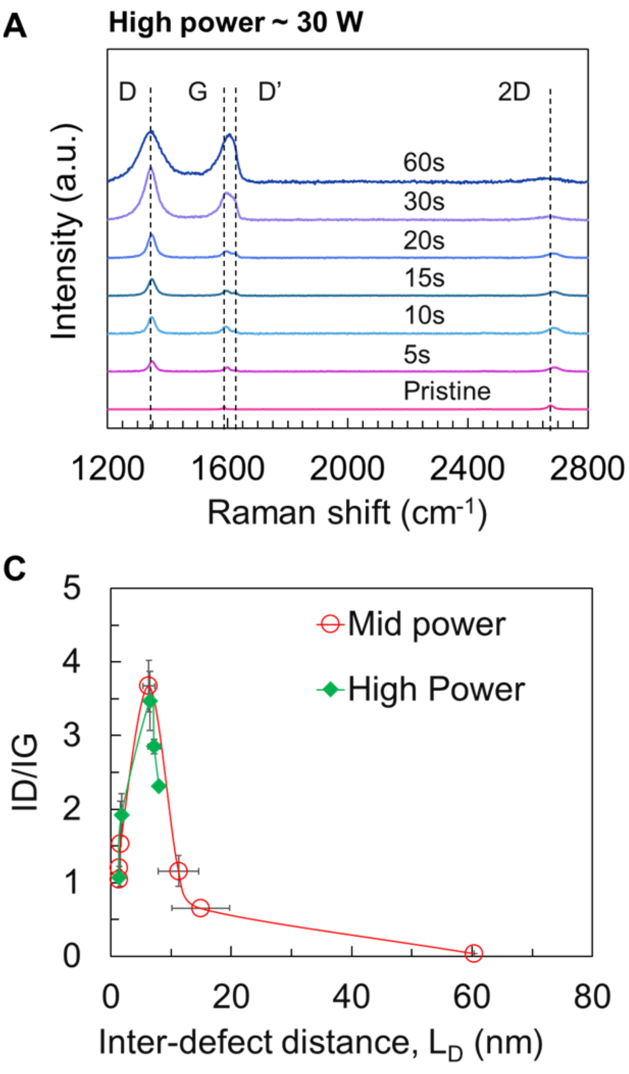

Figure S4. **Raman spectra and analysis.** Raman spectra for CVD graphene transferred on to 300 nm SiO_2/Si wafer before and after Ar plasma exposure at mid power (~11W), high power (~30W) and chamber pressure of ~ 500 mTorr. Ar plasma is performed on CVD graphene transferred to SiO_2/Si wafer via sacrificial PMMA. A) Increasing exposure to Ar plasma results in an increase in the D and D' defect peaks. B) I_D/I_G plot with increasing Ar plasma exposure time computed from Raman spectra in A. C) I_D/I_G plot as a function of inter-defect density, L_D and D) Estimates of defect desnity in graphene with plasma exposure time at different plasma power. Mid power ~11W data is replotted from the main text. In all the cases, plasma chamber pressure was kept constant at ~ 500 mTorr.

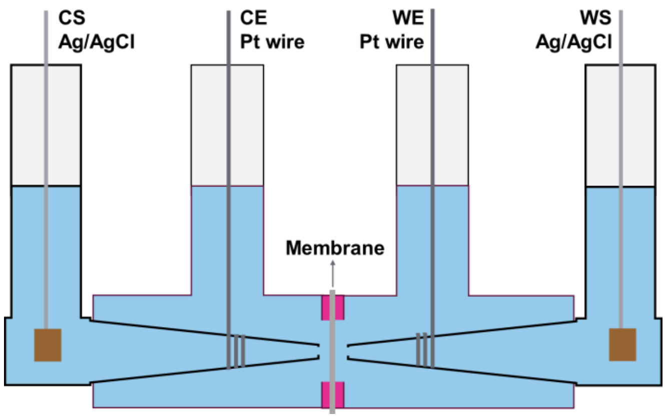

Figure S5. Schematic of custom-built H-cell used for ionic conductance measurements in a four-electrode geometry.

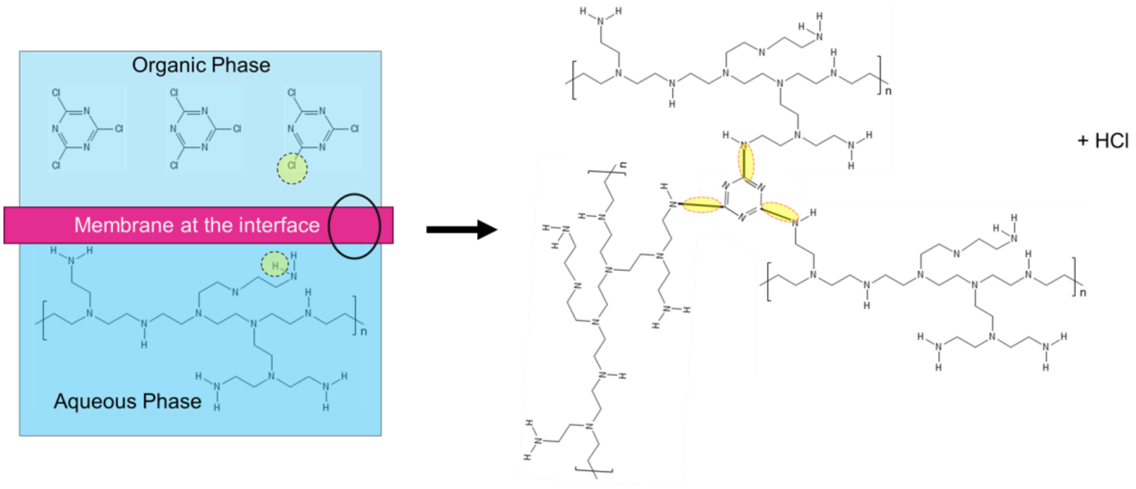

Figure S6. Schematic of acid-resistant interfacial polymerization (IP) to seal defects in CVD graphene on PCTE. Schematic of the reaction at the interface between polyethyleneimine (PEI) and cyanuric chloride acid (see methods for details).

Table S1. Resistance, areal resistance and areal conductance values for PCTE+Gr+Nafion membranes. Active area of the membrane ~ 0.68 cm^2 and 10% porosity of PCTE membranes is used for area normalization.

	Resistance (Ω)	Areal resistance (cm^2)	Solution resistance (Ω cm^2)	Resistance after solution correction (Ω cm^2)	Areal conductance after solution correction (S cm^{-2})
PCTE+Nafion	8.39	0.165	0.128	0.0367	27.3
PCTE+Gr+Nafion	27.7	0.544	0.128	0.416	2.4
PCTE+Gr+Ar 5s+Nafion	14.7	0.289	0.156	0.133	7.5
PCTE+Gr+Ar 20s+Nafion	11.3	0.223	0.156	0.067	15.0
PCTE+Gr+Ar 20s+IP+Nafion	10.5	0.207	0.128	0.079	12.7

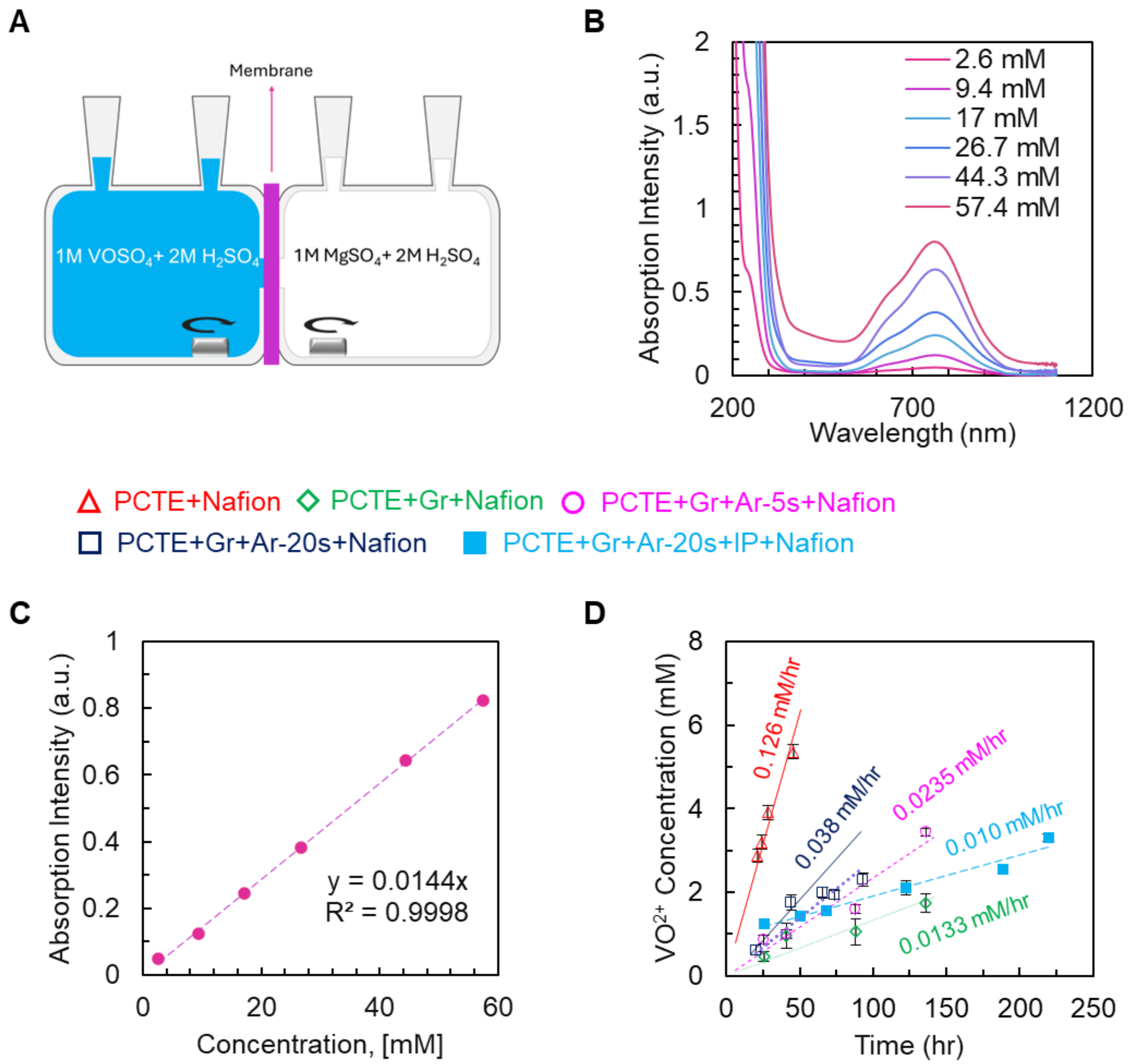

Figure S7. Diffusive transport of VO^{2+} ions (VO^{2+} crossover) through the fabricated membranes measured via UV-vis spectroscopy. A) Schematic of the setup for measuring diffusive crossover of VO^{2+} through the fabricated membranes. Feed side consists of 1M $VOSO_4$ + 2M H_2SO_4 and permeate side of 1M $MgSO_4$ + 2M H_2SO_4. B) UV-vis absorption spectra for known concentrations of VO^{2+} with corresponding C) calibration plots between absorption intensity (peak ~765 nm) and known concentration. D) Plot of VO^{2+} diffusion crossover concentration (mM) as a function of time for all membranes on PCTE supports.

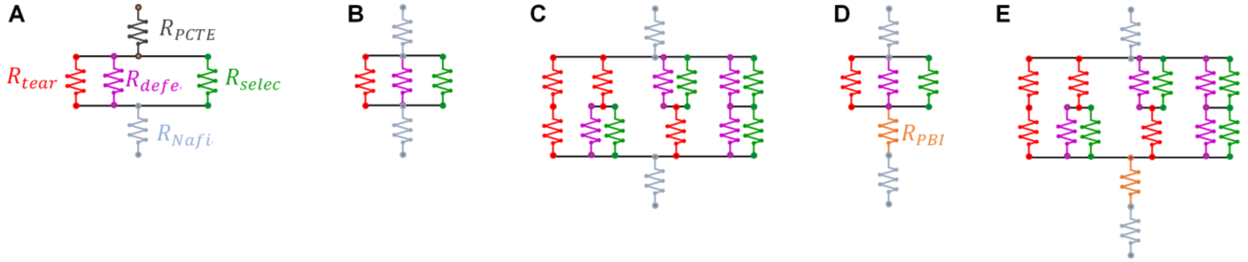

Figure S8. Resistance based transport model depicting the different transport resistances for each membrane configuration. Color coded resistance models for A) PCTE+Gr+Nafion membrane, B) Nafion|Gr|Nafion membrane, C) Nafion|Gr+Gr|Nafion membrane, D) Nafion|Gr+PBI|Nafion membrane and E) Nafion|Gr+Gr+PBI|Nafion membrane. Resistances are color coded by type (R_{PCTE} – dark grey, R_{tear} – red, R_{defect} – magenta, $R_{selective}$ - green, R_{Nafion} – blue, R_{PBI} - orange). Note that weighting factors accounting for the fraction of area corresponding to each branch have been omitted in the diagram but are accounted for in the model.

Table S2. Model parameters to match the measured conductance and crossover

	PCTE+Gr+ Nafion	Nafion|Gr| Nafion	Nafion|Gr+PBI |Nafion	Nafion|Gr+Gr +PBI|Nafion
a [%]	0.05	0.20	0	0
$\overline{D}_{defect}$ [nm]	1.00	0.90	0.16	0.16
$\overline{D}_{selective}$ [nm]	1.20	0.30	0.14	0.07
n_{defect} [cm^{-1}]	2.0×10^8	3.2×10^9	2.0×10^{10}	2.0×10^{10}
$n_{selective}$ (Ar 5 s) [cm^{-2}]	1.0×10^9	4.0×10^{11}	1.5×10^{10}	–
$n_{selective}$ (Ar 20 s) [cm^{-2}]	4.0×10^9	–	1.2×10^{11}	–
$n_{selective}$ (Ar 30 s) [cm^{-2}]	–	–	2.4×10^{13}	4.5×10^{11}

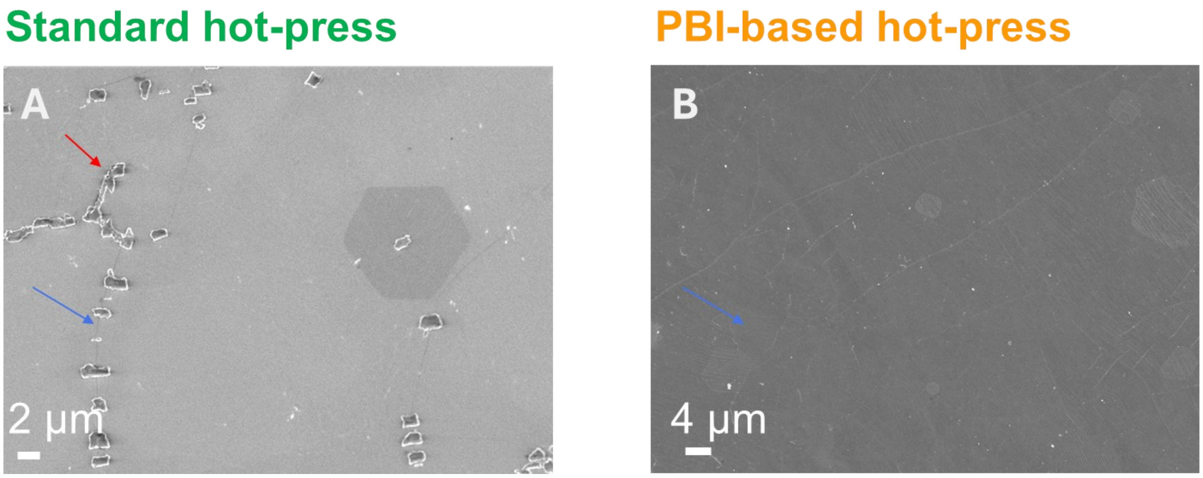

Figure S9. **Graphene transfer to Nafion with and without ~300-320nm of PBI.**
SEM images of CVD graphene transferred to Nafion via A) standard hot-press and B) PBI based hot-press procedures. Defects are present in figure A and are shown via arrows, whereas no such defects are observed for PBI based CVD graphene transfer suggesting higher quality integration/transfer CVD graphene with PBI.

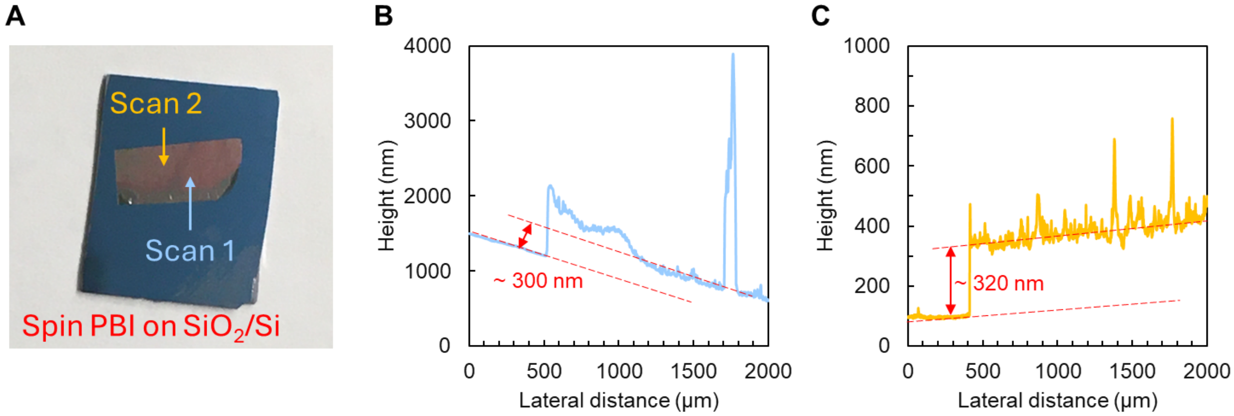

Figure S10. Thickness of spin coated PBI layer measured via profilometry. A) Optical image showing the spin coated PBI transferred on 300 SiO_2/Si wafer. Height profile from two directions measured across the film labelled as B) scan 1 and C) scan 2.

5 wt% PBI was spin coated on Gr+Cu substrate and the Cu was etched, the stack floated on DI water and scooped on 300 nm SiO_2/Si wafer followed by baking at 45°C to remove interfacial water. Measurements were performed using a KLA Tencor P-7 Profilometer.

Table S3. Areal resistance and areal conductance values for N212 and N211 composite membranes. Active area of the membrane is ~ 0.68 cm^2.

	Areal resistance (Ω cm^2)	Areal solution resistance (Ω cm^2)	Areal resistance after solution correction (Ω cm^2)	Areal conductance after solution correction (S cm^{-2})	Conductivity (mS cm^{-1})
N212	0.155	0.1	0.055	18.06	90.3
N212+Gr	0.167	0.1	0.067	15.02	75.1
N212||N212	0.217	0.12	0.095	10.58	105.8
N212|Gr|N212	0.241	0.12	0.119	8.40	84.0
N212|Gr+Gr|N212	0.315	0.12	0.193	5.18	51.8
N212|Gr-Ar 5s+Gr-Ar 5s|N212	0.219	0.12	0.097	10.27	102.7
N211|PBI|N211	0.189	0.12	0.069	14.49	72.5
N211|Gr+PBI|N211	0.347	0.1	0.247	4.05	20.3
N211|Gr+Ar 5s+PBI|N211	0.25	0.1	0.15	6.67	33.4
N211|Gr+Ar 20s+PBI|N211	0.192	0.1	0.092	10.87	54.5
N211|Gr-Ar 30s+PBI|N211	0.167	0.1	0.067	14.93	74.7
N211|Gr-Ar 30s+Gr-Ar 30s+ PBI|N211	0.217	0.1	0.117	8.55	42.8

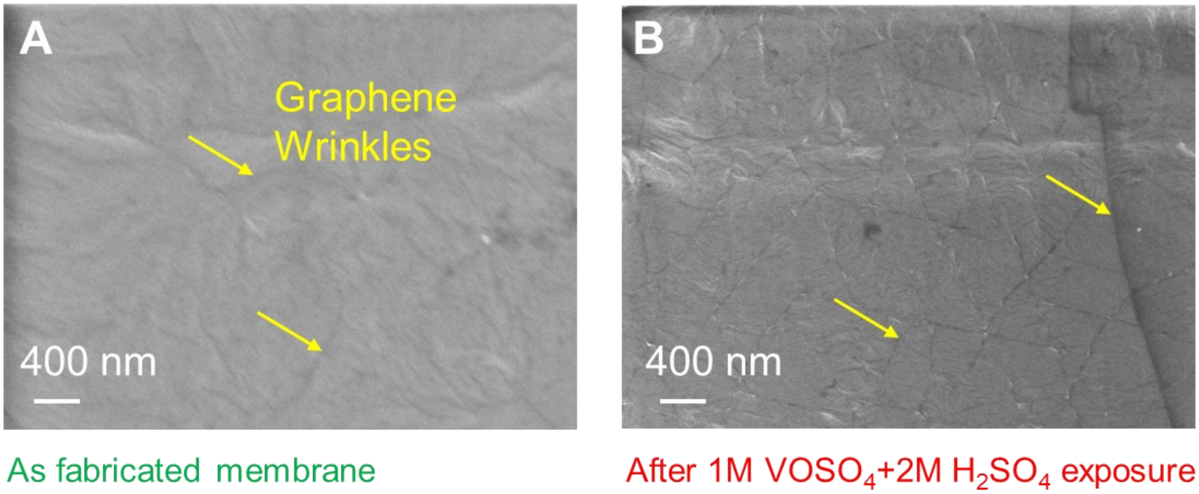

Figure S11. Mechanical stability of graphene after exposure to electrolyte. SEM image of A) as prepared membrane N212|Gr and B) after exposure to 1M $VOSO_4$+2M H_2SO_4 electrolyte. Arrows point to wrinkles present on graphene. N212|Gr membrane is used instead of the sandwich geometry to directly observe the graphene via SEM. Hence, the graphene is not covered with another layer of Nafion on top.

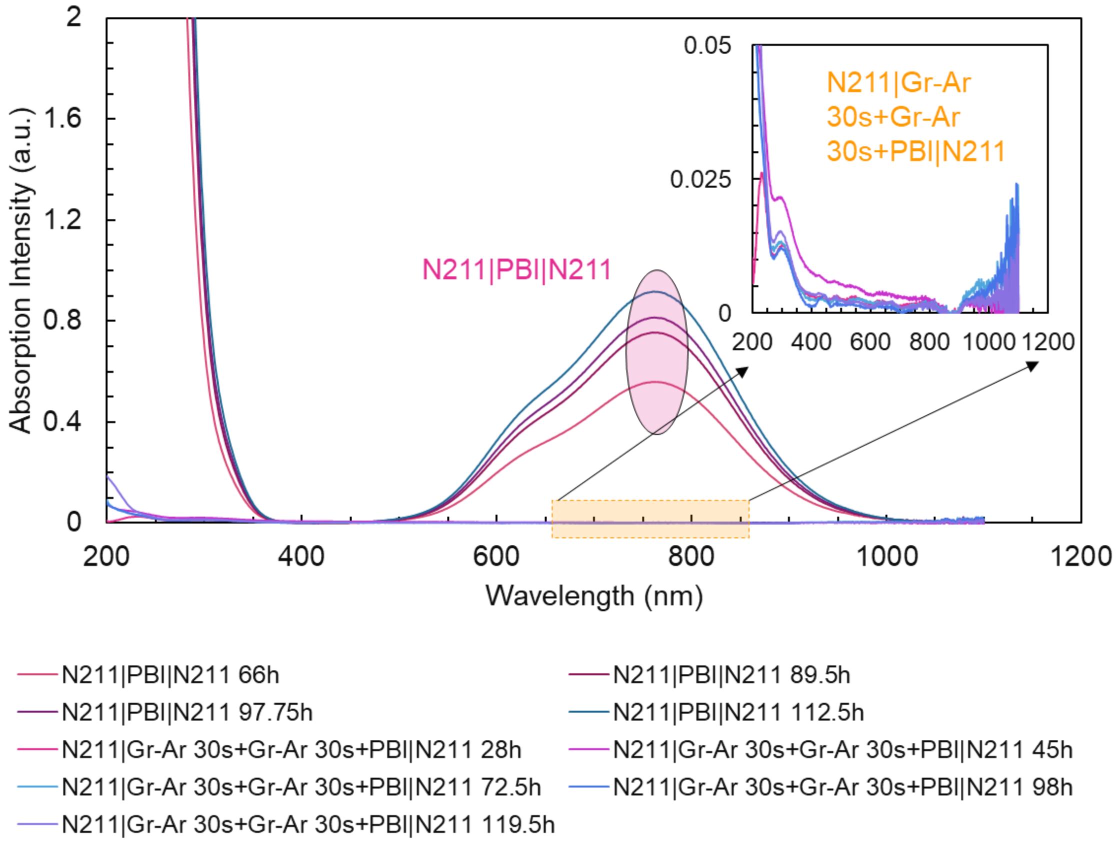

Figure S12. **Diffusive transport of VO^{2+} ions (VO^{2+} crossover) through the Nafion sandwich composite membranes measured via UV-vis spectroscopy.**

UV-vis spectra of VO^{2+} diffusion/crossover as a function of time for N211|PBI|N211 and N211|Gr-Ar 30s+Gr-Ar 30s+PBI|N211 membranes. The N211 | Gr-Ar 30s + Gr-Ar 30s + PBI | N211 membranes shows lower crossover compared to the N211|PBI|N211 control membrane.

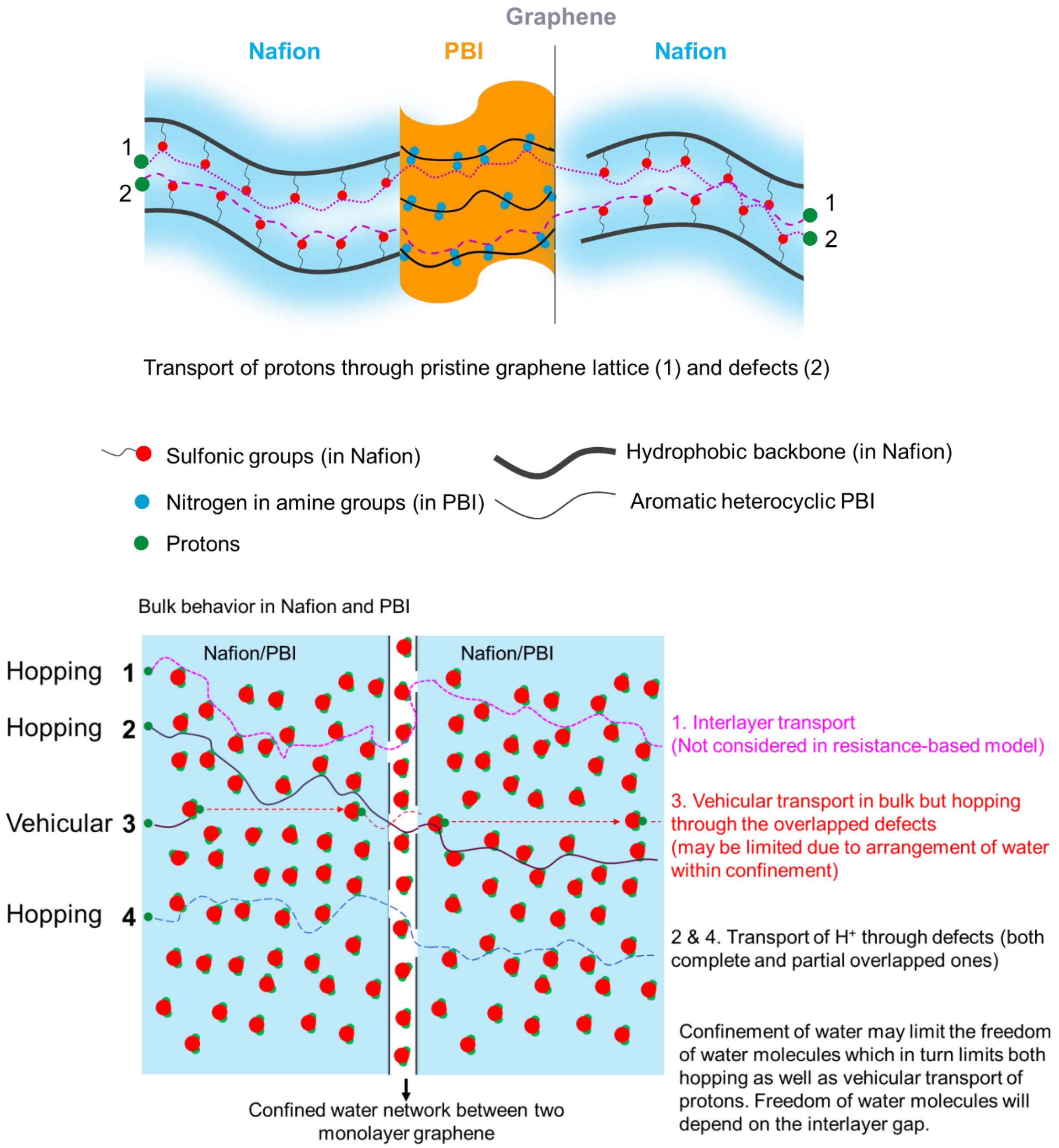

Figure S13. **Proton transport pathways in the fabricated membranes.**

Top – simplistic sketch of proton transport pathway.

Bottom – simplistic sketch of transport of protons through interlayer gap between two stacked monolayers of CVD graphene filled with water. The availability of water and its structural freedom *i.e.* possibility of rearrangement will affect the transport and have not been considered in detail here in this simplistic schematic.

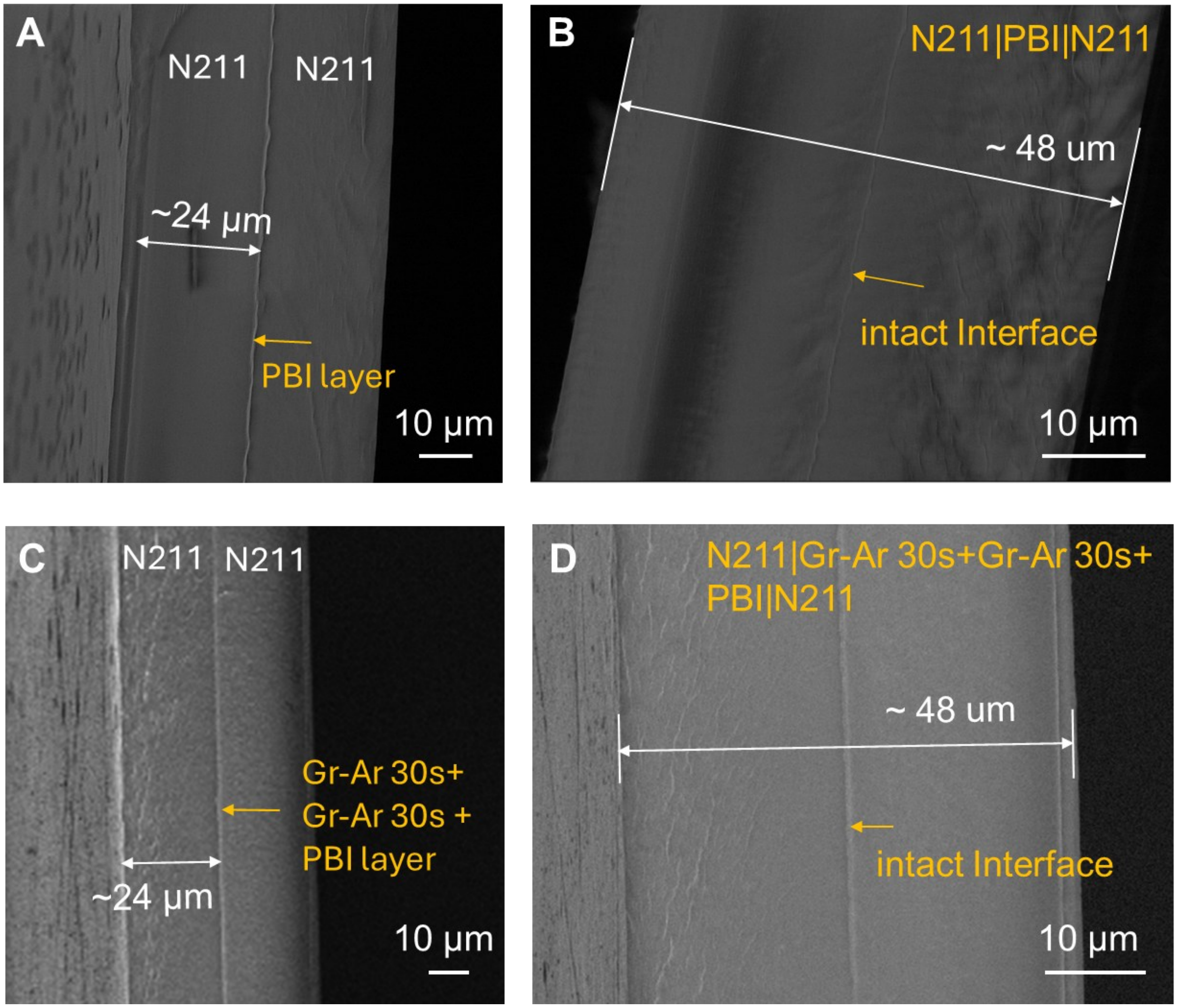

Figure S14. **Cross-section SEM imaging of the Nafion, graphene and PBI composite interface.** SEM image of A,B) N211|PBI|N211 and C,D) N211|Gr-Ar 30s+Gr-Ar 30s+PBI|N211 membrane after proton conductance and VO^{2+} crossover measurements. The cross section was prepared by freezing the membrane by soaking in liquid nitrogen and cracking it under mechanical force to provide a clean interface without smearing effects from cutting with a razor blade.

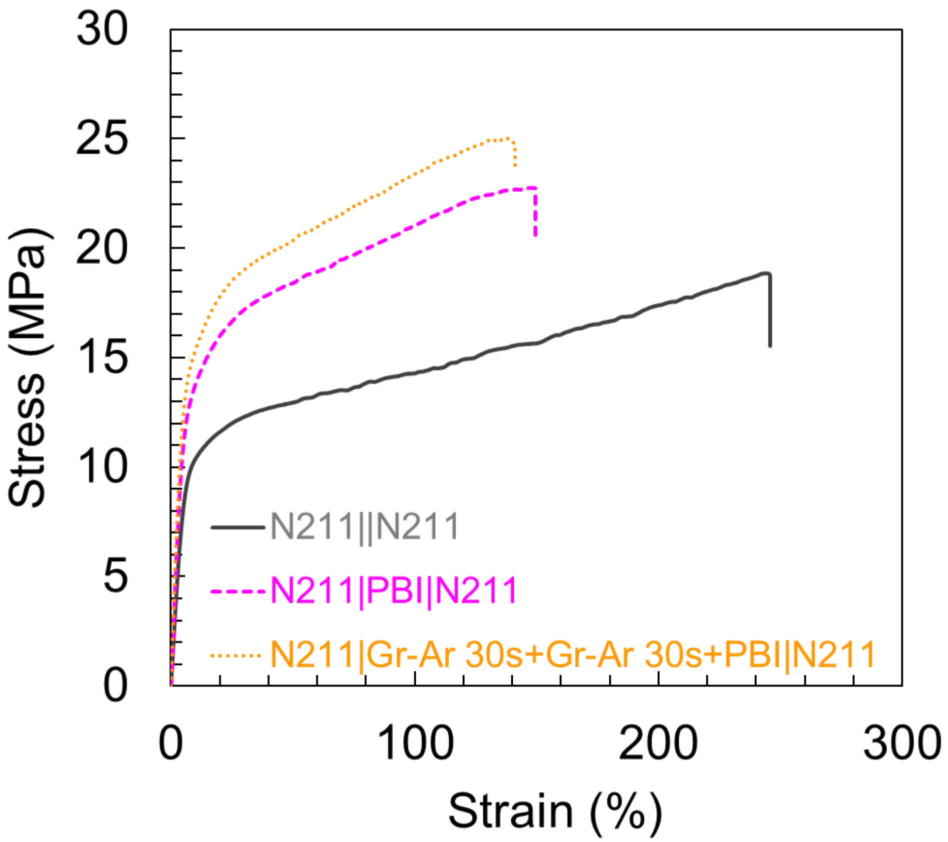

Figure S15. **Mechanical strength of the membranes.** Stress-strain curve for N211||N211, N211|PBI|N211 and N211|Gr-ar 30s+Gr-Ar 30s+PBI|N211 membranes. Mechanical property of the membranes (0.6 × 2 cm) was conducted on a Instron 5944 mechanical testing system with a tensile speed of 5 mm min^{-1}.

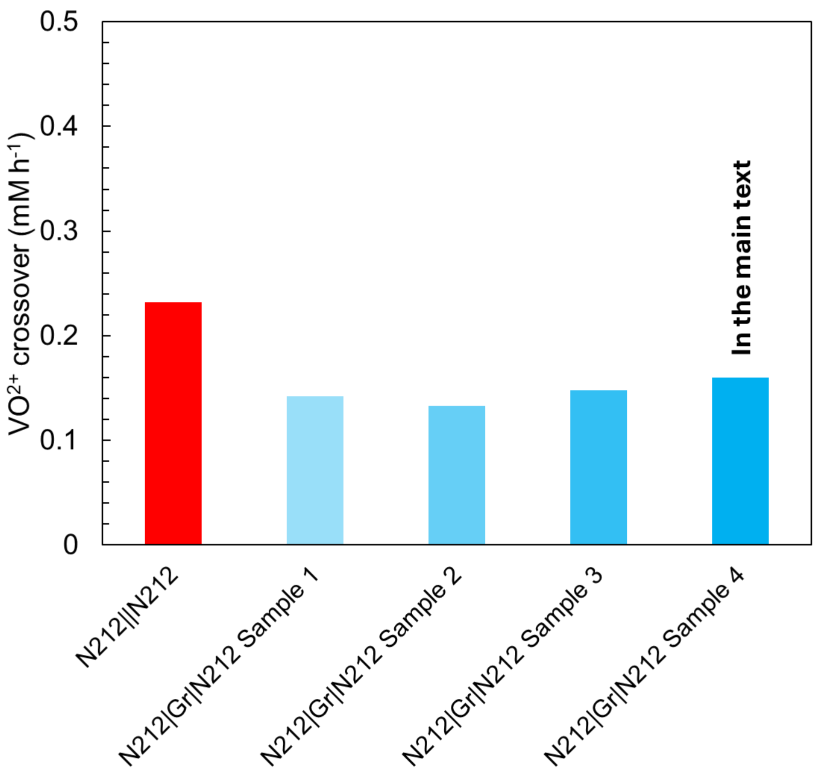

Figure S16. **Reproducibility of the fabricated membranes.**

VO^{2+} crossover measurements for multiple N212|Gr|N212 membranes.

VO^{2+} crossover for 4 distinct N212|Gr|N212 samples (including the one data point discussed in the manuscript). All samples were measured for crossover to confirm the graphene transfer/integration quality and confirmed consistent graphene integration with N212 membranes resulting in crossover reduction to ~0.15±0.01 mM h^{-1}.

Table S4. Permeability of VO^{2+} ions for N212 and N211 composite membranes.
Diffusion measurements are performed using 1M $VOSO_4$+2M H_2SO_4 and 1M $MgSO_4$+2M H_2SO_4 with an active membrane area ~ 0.196 cm^2.

Samples	VO^{2+} crossover ($mM\ h^{-1}$)	Permeability ($cm^2\ min^{-1}$)
N212	0.36	9.94×10^{-7}
N212+Gr	0.23	6.35×10^{-7}
N212‖N212	0.2316	1.28×10^{-6}
N212|Gr|N212	0.14717	8.12×10^{-7}
N212|Gr+Gr|N212	0.066	3.64×10^{-7}
N212|Gr-Ar 5s+Gr-Ar 5s|N212	0.1534	8.47×10^{-7}
N211‖N211	0.407	1.04×10^{-6}
N211|PBI|N211	0.2146	5.47×10^{-7}
N211|Gr+PBI|N211	0.001	2.55×10^{-9}
N211|Gr+Ar 5s+PBI|N211	0.0019	4.84×10^{-9}
N211|Gr+Ar 20s+PBI|N211	0.0035	7.43×10^{-9}
N211|Gr-Ar 30s+PBI|N211	0.158	3.35×10^{-7}
N211|Gr-Ar 30s+Gr-Ar 30s+ PBI|N211	0.0005	1.27×10^{-9}

Table S5. Swelling of the fabricated composite membranes after soaking in water and electrolyte for 24 hours. Membrane thickness was measured at multiple locations for obtaining standard deviations.

Samples	Thickness (dry, µm)	Thickness (wet, µm)	Swelling ratio (through-plane, %)	Swelling ratio (in-plane, %)
	Water			
N211||N211	52.8±1.2	61.7±0.9	16.9±3.7	25.4±1.5
N211|PBI|N211	51.5±0.5	58.7±0.5	14.0±1.8	17.0±3.8
N211|Gr-Ar 30s+Gr-Ar 30s|PBI|N211	53.5±1.1	59.0±0.8	11.7±3.4	12.6±1.6
	1M $VOSO_4$+2M H_2SO_4			
N211||N211	52.8±1.5	57.7±1.2	9.2±4.9	15.0±0.5
N211|PBI|N211	51.5±0.5	55.0±0.8	7.0±2.4	13.1±0.5
N211|Gr-Ar 30s+Gr-Ar 30s|PBI|N211	53.5±1.1	57.7±0.8	6.5±3.4	8.8±1.3

Table S6. Summary of mechanical properties of Nafion PBI composite membranes.

Sample	Breaking strength (MPa)	Elastic modulus (MPa)	Percentage elongation (%)
N211||N211	18.9	170	240
N211|PBI|N211	22.7	240	148
N211|Gr-Ar 30s+Gr-Ar 30s|PBI|N211	24.9	256	134

References:

1. P. R. Kidambi, R. A. Terry, L. Wang, M. S. H. Boutilier, D. Jang, J. Kong, R. Karnik, Assessment and Control of the Impermeability of Graphene for Atomically Thin Membranes and Barriers. *Nanoscale* **2017**, *9*, 8496.

2. P. Chaturvedi, N. K. Moehring, P. Cheng, I. Vlassiouk, M. S. H. Boutilier, P. R. Kidambi, Deconstructing Proton Transport through Atomically Thin Monolayer CVD Graphene Membranes. *J Mater Chem A Mater* **2022**, *10*, 19797.

3. N. K. Moehring, P. Chaturvedi, P. Cheng, W. Ko, A. P. Li, M. S. H. Boutilier, P. R. Kidambi, Kinetic Control of Angstrom-Scale Porosity in 2D Lattices for Direct Scalable Synthesis of Atomically Thin Proton Exchange Membranes. *ACS Nano* **2022**, *16*, 16003.
